# *Eimeria falciformis* secretes extracellular vesicles to modulate proinflammatory response during interaction with mouse intestinal epithelial cells

**DOI:** 10.1186/s13071-022-05364-x

**Published:** 2022-07-08

**Authors:** Joshua Seun Olajide, Ling Xiong, Shunli Yang, Zigang Qu, Xiao Xu, Bin Yang, Jing Wang, Baohong Liu, Xueting Ma, Jianping Cai

**Affiliations:** 1 State Key Laboratory of Veterinary Etiological Biology, Key Laboratory of Veterinary Parasitology of Gansu, Lanzhou Veterinary Research Institute, Chinese Academy of Agricultural Sciences, Lanzhou, 730046 China; 2 Centre for Distance Learning, Obafemi Awolowo University, Ile-Ife, Nigeria

**Keywords:** *Eimeria falciformis*, Secretome, Extracellular vesicles, Inflammasomes, Pyroptosis, Host-parasite interactions

## Abstract

**Background:**

Protozoan parasite secretions can be triggered by various modified media and diverse physicochemical stressors. Equally, host-parasite interactions are known to co-opt the exchange and secretion of soluble biochemical components. Analysis of *Eimeria falciformis* sporozoite secretions in response to interaction with mouse intestinal epithelial cells (MIECs) may reveal parasite secretory motifs, protein composition and inflammatory activities of *E. falciformis* extracellular vesicles (EVs).

**Methods:**

*Eimeria falciformis* sporozoites were allowed to interact with inactivated MIECs. Parasite secretions were separated into EV and vesicle-free (VF) fractions by discontinuous centrifugation and ultracentrifugation. Secreted EVs were purified in an iodixanol density gradient medium and the protein composition of both EV and VF fractions were analyzed by liquid chromatoraphy-tandem mass spectroscopy. The inflammatory activities of *E. falciformis* sporozoite EV on MIECs were then investigated.

**Results:**

During the interaction of *E. falciformis* sporozoites with inactivated MIECs, the parasite secreted VF and vesicle-bound molecules. *Eimeria falciformis* vesicles are typical pathogenic protozoan EVs with a mean diameter of 264 ± 2 nm, and enclosed heat shock protein (Hsp) 70 as classical EV marker. Refractile body-associated aspartyl proteinase (or eimepsin), GAP45 and aminopeptidase were the main components of *E. falciformis* sporozoite EVs, while VF proteins include Hsp90, actin, Vps54 and kinases, among others. Proteomic data revealed that *E. falciformis* EV and VF proteins are aggregates of bioactive, antigenic and immunogenic molecules which act in concert for *E. falciformis* sporozoite motility, pathogenesis and survival. Moreover, in MIECs, *E. falciformis* EVs induced upregulation of gene expression and secretion of IL-1β, IL-6, IL-17, IL-18, MCP1 as well as pyroptosis-dependent caspase 11 and NLRP6 inflammasomes with the concomitant secretion of lactate dehydrogenase.

**Conclusions:**

*Eimeria falciformis* sporozoite interaction with MIECs triggered the secretion of immunogenic and antigenic proteins. In addition, *E. falciformis* sporozoite EVs constitute parasite-associated molecular pattern that induced inflammatory response and cell death. This study offers additional insight in the secretion and protein composition of *E. falciformis* secretomes as well as the proinflammatory functions of *E. falciformis* sporozoite EVs.

**Graphical Abstract:**

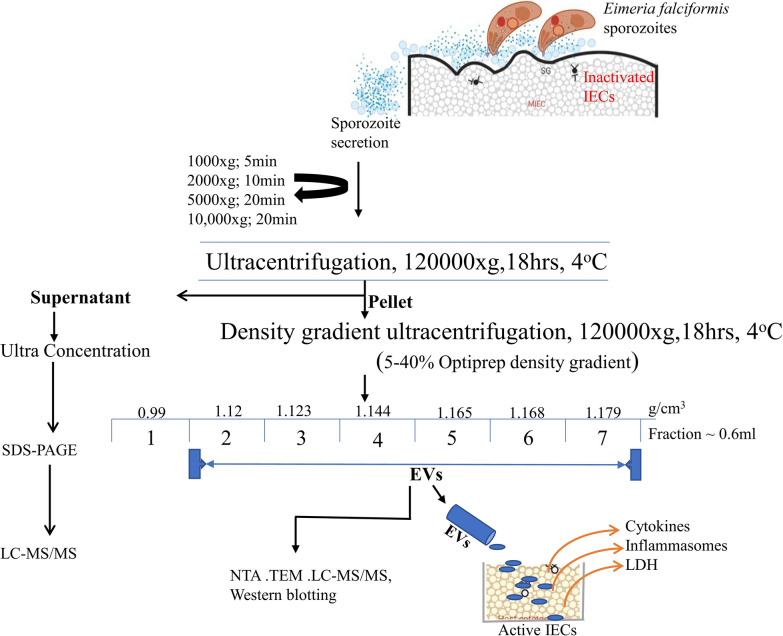

**Supplementary Information:**

The online version contains supplementary material available at 10.1186/s13071-022-05364-x.

## Background

Host-parasite interaction is a dynamic and phenomenal feature of parasitism. It co-opts parasite development and pathogenesis by means of cellular signaling, inflammation, cell death and, essentially, the excretion or secretion of molecules. In general, parasite secretions (or secretomes) are formed along several secretory pathways and released during the host-parasite interplay [1]. Previous studies have described and characterized the secretion of extracellular vesicles (EVs) and vesicle-free (VF) molecules from parasitic protozoa and helminths [2, 3] as well as from parasite-infected host cells [4]. Similarly, there have been several studies on the secretion, characterization and proteomic profiling of *Eimeria* sporozoite proteins triggered by thermal, chemical and mechanical stressors [5]. In addition, VF molecules have been induced and characterized from protozoan parasites in various modified mediums and by physicochemical stressors [5, 6]. Pathogenic protozoan EVs activated by chemical stressors and serum-starved media have also been characterized and  profiled [7]. A few studies have also indicated that host-parasite interactions could induce the secretion of vesicle-bound and VF molecules. In this regard, the formation of parasite secretomes could occur prior to [8] or during protozoan parasite attachment or invasion of the host cell [3, 8–10], but the description of distinct parasite secretions during interactions with the host cell, and characterization of such parasite-derived secretomes have not been demonstrated.

Extracellular vesicles are heterogeneous, sub-cellular, lipid-bound vesicles formed during pathophysiological processes and host-pathogen interactions [11]. Based on biogenesis and size, EVs are broadly classified as exosomes, microvesicles/microparticles and apoptotic bodies [7, 11–15]. EVs usually enclose soluble components of cellular origin, such as metabolic intermediates, glycoconjugates, lipids, proteins and nucleic acids [14]. Functionally, protozoan parasite EVs can deliver their cargoes to mediate parasite motility and development, differentiation, cytoadherence and pathogenesis [16–20]. During host–pathogen interaction, EVs are pivotal in such processes as intercellular communication, horizontal gene transfer, disease biomarker, antigen presentation, immune response and host cell death [14, 15, 21–23].

Typically, *Eimeria* spp. are obligate intracellular apicomplexan parasites [16]. More specifically, *Eimeria falciformis* naturally parasitizes caecal epithelial cells of wild and laboratory mice, causing catarrhal enteritis, hemorrhage and epithelial sloughing [17, 18]. The life-cycle of *E. falciformis* is similar to that of other coccidian parasites, with the sporozoites, after excystation from the sporocyst, actively migrate through the mice gut before invading the host intestinal epithelial cells (IECs) [17, 18, 25]. In general, parasitization of the host cells by apicomplexan parasites involves gliding motility, cellular attachment, invasion and spontaneous formation of excretory/secretory products with which apicomplexans modulate host cell responses [10, 26–28]. However, *E. falciformis* sporozoite proteins secreted during interaction with the host IECs have not been described [5]. Also, the secretion of parasite-derived EVs has not been characterized in *Eimeria* spp. [7], nor have the inflammatory responses of host IECs, such as the secretion of cytokines and formation of inflammsome complexes, to *Eimeria*-derived EVs.

In this study, *E. falciformis* sporozoites were allowed to interact with inactivated mouse IECs (MIECs), and *E. falciformis* sporozoite VF and EV fractions were separated, purified and analyzed. In addition, MIECs were stimulated with *E. falciformis* sporozoite EVs (*Ef*SEVs) to determine differential regulation and expression of inflammatory cytokines (interleukin [IL]-1β, IL-6, IL-17, IL-18), the chemokine monocyte chemoattractant protein 1 (MCP1) and pyroptosis-dependent caspase 11 and nucleotide-oligomerization domain (NOD)-like receptor pyrin 6 (NLRP6) inflammasomes, and to reveal underlying synergy between MIEC inflammatory response and lethal activity of *Ef*SEVs.

## Methods

### Parasite propagation

Parasite propagation was carried out as described earlier [17, 29, 30] with modification. In brief, 100 specific pathogen-free Balb/c mice aged between 6 and 8 weeks were raised in plastic cages placed in a pathogen-free room with adequate supply of food and sterile water. Each mouse was orally inoculated with 50 µl of 2 × 10^5^ *E. falciformis* sporulated oocysts/ml phosphate-buffered saline (PBS). Oocysts shed in feces between 7- and 11-day post-infection were recovered from homogenized fecal solution, washed through a metal strainer and purified by salt flotation. Purified oocysts were washed and suspended as appropriate in 2.5% potassium dichromate followed by incubation at 28 °C and 120 rpm for approximately 10 days. Sporulated oocysts were stored at 5 °C and used within 30 days of storage.

### Purification of *E. falciformis* sporozoites

*Eimeria falciformis* sporozoites were excystated and purified as earlier reported [31–33]. Briefly, *E. falciformis* sporulated oocysts were washed in PBS to remove potassium dichromate through discontinuous centrifugation. Purified oocysts were precipitated in 40 ml of antibiotic–antimycotic diluent (1 part dilutent per 100 ml) (Solarbio, Beijing China). *Eimeria falciformis* sporulated oocysts were then suspended in 5% (v/v) sodium hypochlorite for 20 min on ice with intermittent stirring, and  the oocysts were washed in PBS. The hypochlorite-treated sporulated oocysts were then vortexed with an equal volume of glass beads (diameter: 0.5 mm) for 3 min to release sporocysts and thereafter incubated with 0.75% (w/v) sodium taurodeoxycholate and 0.25% (w/v) trypsin in PBS for 1 h at 37 °C and 200 rmp for sporozoite excystation. The final purification step was vacuum filtration of the *E. falciformis* sporozoites [19, 32].

### Cell and parasite culture

Mouse intestinal epithelial cells were cultivated in complete medium (Dulbecco’s modified Eagle’s medium [DMEM; Sigma-Aldrich, St. Louis, MO, USA) supplemented with 10% fetal bovine serum (FBS) (Sigma-Aldrich) (v/v) and 1% antibiotic diluent (Solarbio, Beijing, China)[26]. After confluence was reached, approximately 1 × 10^6^ MIECs were plated with complete medium and incubated overnight. To inhibit exosome secretions [27], MIECs were inactivated with 4% paraformaldehyde (Solarbio, Beijing, China) for 20 min at room temperature and washed 5 times with sterile PBS. Inactivated MIECs were re-cultured in Exo-clear cell growth medium (System Biosciences, Palo Alto, CA, USA) followed by inoculation of 2 × 10^6^ freshly excysted *E. falciformis* sporozoites [28]. The parasites were maintained in the culture plates for 18 h [30] at 37 °C in a 5% CO_2_ humidified incubator. A non-treated control (NC) experiment was prepared in parallel in which inactivated MIECs were incubated as described above, but without *E. falciformis* sporozoites.

### Separation of *E. falciformis* sporozoite secretomes

A 350-ml sample of culture supernatant was harvested without disrupting the MIEC monolayer and centrifuged at 4 °C sequentially at: 1000 *g*, 5 min; 2000 *g*, 10 min; 5000 *g*, 20 min; 10,000 g, 30 min. The supernatant was then transferred into polycarbonate ultracentrifugation tubes (Thermo Fisher Scientific, Waltham, MA, USA) and centrifuged at 120,000 *g* for 18 h in a Sorvall WX+ Ultra Series centrifuge (T-890 fixed angle rotor, k-factor 25.1; Thermo Fisher Scientific). The resulting supernatant from the ultracentrifugation was subjected to ultrafiltration through a 0.22-µm Corning sterile filter (Corning, Corning, NY, USA) at 4 °C under gravity, followed by 10-fold concentration using an Ultra 10 kDa cut-off membrane (Amicon Ultrafiltration System; MilliporeSigma, Burlington, MA, USA) and stored at − 80 °C as the *E. falciformis* sporozoite VF fraction [31]. The cell culture supernatant from the NCs was prepared in the same manner.

The resulting pellets from the ultracentrifugation process were pooled, reconstituted in 8 ml PBS (pH 7.2) and resolved on discontinuous iodixanol gradients solutions prepared by diluting OptiPrep™ density gradient (ODG) medium (Sigma-Aldrich) in 60% (v/v) aqueous iodixanol with sterilized 0.3 M sucrose/10 mM Tris, pH 7.2 [32, 33]. The gradient was formed by adding 1.6 ml each of 40, 20, 10 and 5% iodixanol solution into a 9-ml polycarbonate tube (Thermo Fisher Scientific) and 1 ml of *Ef*SEV suspension was overlaid and  subjected to ultracentrifugation for 20 h at 120,000 *g* and 4 °C in a Sorvall WX+ Ultra Series centrifuge (T-890 fixed angle rotor, k-factor 25.1; Thermo Fisher Scientific). Seven gradient layers were collected, resuspended in PBS (pH 7.2) and washed twice for 4 h at 120,000 *g* and 4 °C. *Ef*SEV pellets were pooled, concentrated in 400 µl of PBS and stored at − 80 °C. Likewise, NC pellets were collected and washed appropriately. The density of *Ef*SEVs was measured from the corresponding ODG layer after appropriate dilution at an optical density of 244 nm [32, 33] with a UV–Visible spectrophotometer (Biomates 3S; Thermo Fisher Scientific) (Additional file 6 : Data set).

### Transmission electron microscopy

For transmission electron microscopy (TEM), 10 μl of *Ef*SEVs was added to the a formvar carbon film-coated copper grid for 3 min. Excess liquid was removed with filter paper followed by staining with 3% phosphotungstic acid [34, 35]. An equal volume of NC particles was also stained. The grids were observed in model HT770 transmission electron microscope (Hitachi, Tokyo, Japan).

### Nanoparticle tracking analysis

The size of *Ef*SEVs was determined using the Zetasizer Nano-Zs instrument (Malven, Worcestershire, UK). Briefly, 10 μg of *Ef*SEVs and an equal volume of NC pellets were dissolved in an appropriate volume of PBS to make a total volume of 1 ml in each case. The size and distribution of the particles were measured in triplicate [13].

### Sodium dodecyl sulfate-polyacryamide gel electrophoresis and liquid chromatography-tandem mass spectrometry

The protein components of the *Ef*SEV and VF fractions were quantified using the BCA kit (Solarbio, Beijing, China). An equivalent of 10 µg of *Ef*SEV and VF fractions, as well as the NC and NCs were separately mixed with lysis buffer (Solarbio, Beijing, China) according to the manufacturer’s instruction, heated in a water bath at 100 °C for 10 min and resolved by 12% sodium dodecyl sulphate–polyacrylamide gel electrophoresis (SDS-PAGE) [35, 36] at 100 V in a Mini-Cell electrophoresis system (BioRad Laboratories, Hercules, CA, USA). Following electrophoresis, the SDS-PAGE gel was stained with a Coomassie blue stain kit. Subsequently, the *Ef*SEV and VF protein gel lanes were excised and pooled for mass spectrometry (MS).

Liquid chromatography-tandem MS (LC–MS/MS) was performed by the Lu-Ming Biotech Co., Ltd. (Shanghai, China). In brief, excised protein gel lanes were dehydrated and dried in a vacuum. Gel particles were reduced in 10 mM DTT/25 mM NH_4_HCO_3_ followed by treatment with alkylation buffer and recovered in 25 mM NH_4_HCO_3_. Reduced gel particles were digested in 20 μl of 0.02 μg/μl trypsin in 25 mM NH_4_HCO_3_. The supernatant was treated with 50 μl of 5% formic acid/67% acetonitrile followed by elution in 5% formic acid/67% ACN/H_2_O. After drying using low-pressure centrifugation, the sample was re-dissolved in 40 µl of 0.1% formic acid and centrifuged through a column. The digested peptide elute was adjusted to pH 7 by H_3_PO_4_ and the resulting peptides were lyophilized in 150 μl of 100% methanol. MS analyses were performed with a Q-Exactive mass spectrometer (Thermo Fisher Scientific) equipped with Nanospray Flex source (Thermo Fisher Scientific). MS/MS spectra were obtained with a resolution of 17, 500 and an AGC target of 2e^5^ at a maximum injection time of 50 ms. The Q-E dynamic exclusion was set at 15.0 s in positive mode.

### Protein database search and bioinformatics

MaxQuant, a quantitative proteomics software package, was used to search peptide sequences against *Eimeria* species, *Toxoplasma gondii* and *Plasmodium falciparum* proteins in UniProt. Trypsin digestion specificity was used to search databases. For protein quantification, MS1 and MS2 tolerance were set at 10 ppm and 0.02 Da, respectively. Gene ontology (GO) and Kyoto Encyclopedia of Genes and Genome (KEGG) analyses were also performed.

### Western blot

*Ef*SEV and VF protein concentrations were measured using a BCA kit (Solarbio, Beijing, China). An equivalent volume (10 µg) of *Ef*SEV, VF, NC and NCs was separately mixed with 4× Loading Buffer (Solarbio, Beijing, China) and vortexed. The mixture was heated in a water bath at 100 °C for 10 min. The protein components were resolved in a 12% SDS-PAGE gel, and the protein bands were transferred to a polyvinylidene difluoride membrane (Merck-Millipore, Germany). After 1 h of blocking with 0.01% Tween-20 in PBS (PBST) containing 5% skimmed milk, the membrane was incubated with anti-heat shock proteins 70 kDa (Hsp70; Proteintech Group Inc., Rosemont, IL, USA) overnight at 4 °C. Thereafter, the membrane was washed in PBST and incubated with horseradish protein-conjugated antibodies (Proteintech Group) for 1 h at room temperature. The membrane was treated with the WesternBright™ ECL enhanced chemiluminescent substrate kit (Advansta Inc., San Jose, CA, USA) according to the manufacturer’s instruction and visualized using the Luminescent Image Analyzer Amersham Imager 600 series (GE Co., Boston, MA, USA).

### Multiplex protein microarray

The MIECs were plated at a density of 1 × 10^6^ in complete medium and incubated overnight at 37 °C and 5% C0_2_. Plated MIECs were subsequently re-cultured in DMEM supplemented with 10% exosome-depleted fetal bovine serum [37] and stimulated with 10, 30, 50 and 100 µg of *Ef*SEVs and PBS (0 µg of *Ef*SEVs) was used as experimental control treatment. Similarly, an equal density of MIECs was treated with 50 µg of *Ef*SEVs for 0, 6, 12 and 18 h. Cell culture supernatants were collected for protein microarrays. IL-1β, IL-6, IL-17, IL-18 and MCP1 proteins were quantified by QAM-INF-1 (Quantibody® Mouse Inflammatory Array 1 Kit; RayBiotech, Peachtree Corners, GA, USA) [38]. Analyses were done in triplicate, and targeted cytokines were tested in quadruplicate. Each cytokine was quantified as mean fluorescence using the InnoScan 300 Microarray Scanner (Innopsys, Carbonne, France) at a wavelength of 532 nm and a resolution of 10 µm. Data were analyzed using QAM-INF-1 analysis software (RayBiotech).

### RNA extraction and quantitative PCR

RNA was extracted from *Ef*SEV-stimulated MIECs using TRIzol reagents (Invitrogen, Thermo Fisher Scientific). In parallel, the same density of MIEC was treated with NC particles. A 1-µg aliquot of total RNA was reverse transcribed with the Primescript™ RT Reagent Kit (Takara Bio Inc., Shiga, Japan) according to the manufacturer’s instructions. PCR primers for IL-1β, IL-6, IL-17, IL-18, MCP1, caspase 11 and NLRP6 were synthesized by Tsingke Biological Technology (Xi’an, China) (Additional file 5). Real-time quantitative PCR (qPCR) was performed using TB Green pre-mix Ex Taq™ II (Takara Bio Inc.) and the CFX98 real-time PCR detection system (Bio-Rad, Hercules, CA, USA). Each reaction tube contained 1 μl each of forward and reverse primers, 2 μl of cDNA template, 10 μl of TB Green and 6 μl of nuclease-free water. Assays were performed in triplicate, and data were normalized by using β-actin as a reference gene.

### Lactate dehydrogenase assay

Approximately 1 × 10^6^ MIECs in DMEM supplemented with 10% exosome-depleted FBS were stimulated with 50 µg of *Ef*SEVs for 0, 6, 12, 18 and 24 h. Likewise, 10, 30, 50, and 100 µg of *Ef*SEVs were used to stimulate MIECs and PBS (0 µg of *Ef*SEVs) as experimental control treatment. Also, an equivalent volume of NC particles was used as a negative control for *Ef*SEV time- and dose-dependent production of lactate dehydrogenase (LDH) by MIECs [39, 40]. The culture plates were incubated at 37 °C and 5% CO_2_. LDH was measured by Chekine™ LDH assay kit (Abbkine, Wuhan, China) as instructed in the product manual. Absorbance was measured at 450 nm on a SpectraMax M5e Multi-Mode Microplate Reader (Molecular Devices, LLC, San Jose, CA, USA).

### Statistical analyses

Data were expressed as the mean with the standard deviation and analyzed by one-way analysis of variance. Turkey’s test was used for multiple comparisons. Linear regression analysis was performed using SPSS software version 21 (SPS IBM Corp., Armonk, NY, USA). Charts were generated in GraphPad Prism 7 (GraphPad Software, San Diego, CA, USA). Differences among dose- and time-dependent activities of *Ef*SEVs on MIECs relative to the experimental control were considered to be statistically significant at *P < 0.05, **P < 0.001 and ***P < 0.0001.

## Results

### *E. falciformis* sporozoite secretomes during interaction with MIECs

**Fig. 1:**
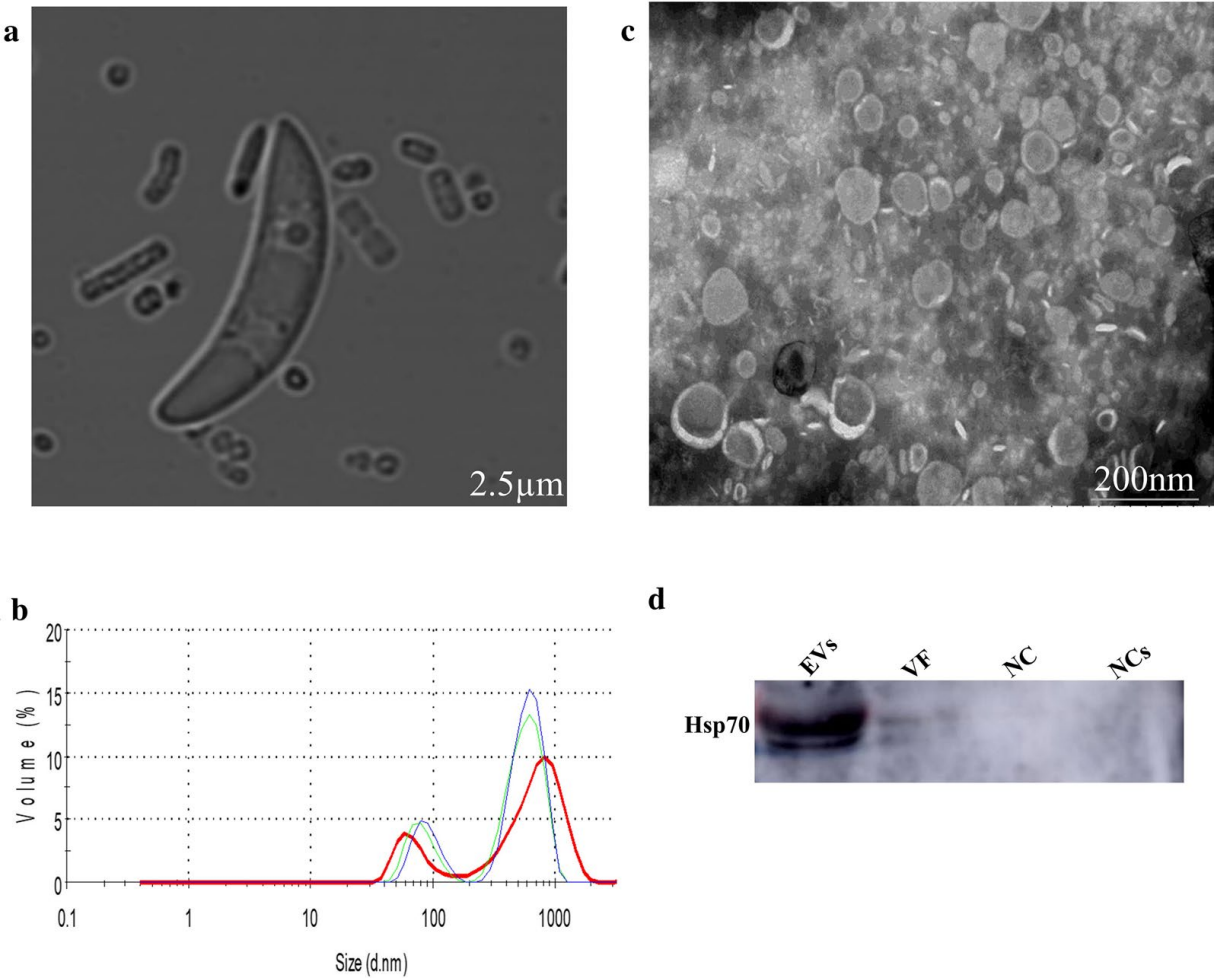
*Eimeria falciformis* sporozoite and derived EVs.** a** *E. falciformis* sporozoite after excystation from sporocyst.** b** Bar graph from NTA analysis of 10 µg *Ef*SEVs in 1 ml of PBS. *Ef*SEV mean diameter was 246 ± 2 nm.** c** TEM image of *Ef*SEV processed through negative staining with 3% phosphotungstic acid.** d** Western blot analysis of *Ef*SEVs using anti-Hsp70. Abbreviations:* Ef*SEV, *E. falciformis* sporozoite EVs; EVs, extracellular vesicles; Hsp70, heat shock protein 70; NC, non-treated control particles; NCs, non-treated control supernatant; TEM, transmission microscopy; VF, vesicle-free (see Additional files 1–5)

The *E. falciformis* sporozoite (Fig. 1a) is the motile, invasive stage of the parasite which navigates through the host gut before invading the host cell. The fractions of *E. falciformis* sporozoite secretome were separated from the culture medium by serial centrifugation and ultracentrifugation steps, and then the scereted EVs were characterized by nanoparticle tracking analysis (NTA) and TEM. Also, *Ef*SEV and VF protein compositions were identified by electrophoresis (Additional file 5) and analyzed by MS (Additional file 6). Our findings revealed that *Ef*SEVs had a buoyant density ranging from 1.12 to 1.179 g/cm^3^ distributed between the second and the seventh ODG layers. The particles from NC had a buoyant density that ranged between 0.57 and 1.103 g/cm^3^ (Additional file 5). NTA analysis revealed that *Ef*SEVs were a heterogeneous population of EVs with a diameter of approximately 37.84–1,281 nm (Fig. 1b). TEM observations indicated that *Ef*SEVs were spherical and circular in shape, similar to *Toxoplasma gondii* and *Neospora caninum* EVs [35, 41] (Fig. 1c). In addition, TEM revealed that NC particles (Additional file 2) did not contain secreted vesicles, indicating that the observed EVs were formed during *E. falciformis* sporozoite interaction with MIECs. Additionally, the vesicular property of *Ef*SEVs and depletion of protein in the non-treated control experiment were also confirmed by targeting Hsp70 in *Ef*SEVs, VF, NC, and NCs for western blotting (Fig. 1d).

### Protein composition of *E. falciformis* sporozoite EV and VF fractions

**Fig. 2:**
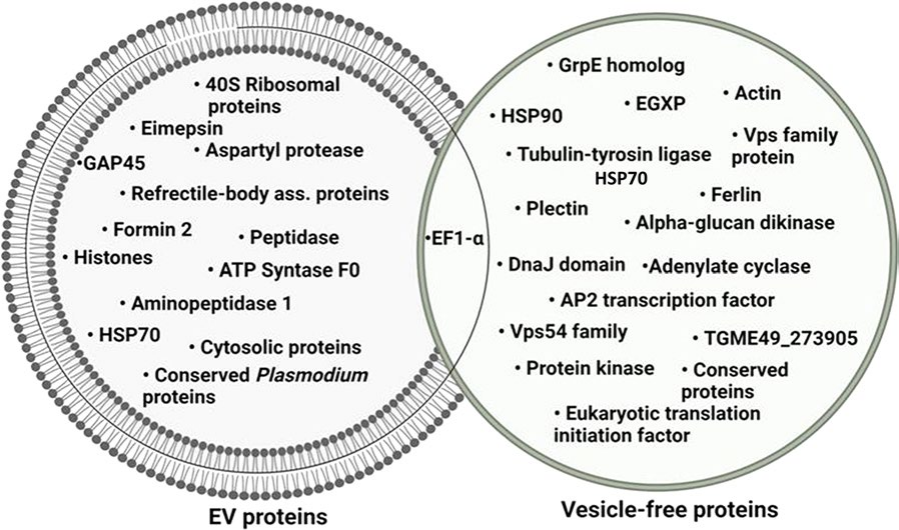
*Ef*SEV and VF proteins identified by mass spectrometry. The list and other information on the identified proteins are provided in Additional file 5

**Fig. 3:**
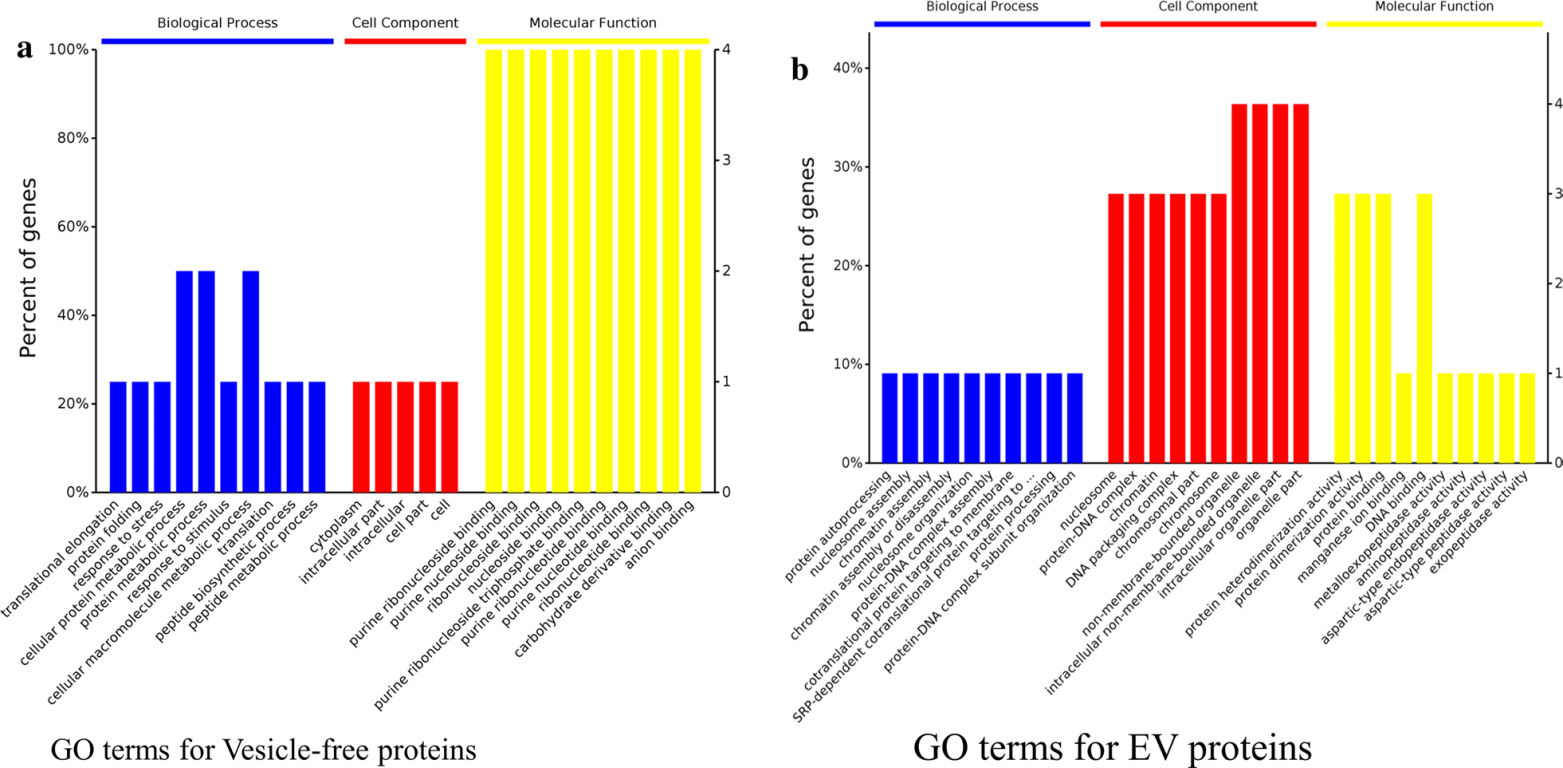
GO annotation of identified *Ef*SEV and VF proteins.** a** GO terms for *E. falciformis* VF proteins,** b** GO terms for *Ef*SEV proteins. See Additional file 5. Abbreviations: GO, Gene ontology

Electrophoretic analyses of the *Ef*SEV and VF fractions showed that *E. falciformis* sporozoites secreted proteins as components of its secretomes (Additional file 6) during interaction with inactivated MIECs. The protein composition of the *Ef*SEV and VF fractions were analyzed by LC–MS/MS. MS/MS spectra were searched in the protein database for avian and bovine *Eimeria* species, *T. gondii* and *P. falciparum* because of the intractable genome of *E. falciformis* [9]*.* Forty-two proteins were identified from the *E. falciformis* sporozoite VF fraction (Additional file 6), including actin (putative), kinases, heat shock proteins (Hsp 70 and Hsp90), vacuolar protein sorting (Vps) 54, elongation factor 1 alpha (EF-1α), early gametocyte enriched phosphoprotein (EGXP), as well as some *P. falciparum* and *T. gondii* orthologous proteins (Fig. 2; Additional file 6). GO terms for *E. falciformis* sporozoite VF protein are shown in Fig. 3a.

Thirty-seven proteins were identified from *Ef*SEVs along with other unidentified protein peptides (Fig. 2; Additional file 6). Identified *Ef*SEV proteins include refractile body (RB)-associated proteins, Hsp70, EF-1α, cytosol aminopeptidase (putative), aspartyl proteinase (or eimepsin) and proteases. Other proteins from *Ef*SEVs were gliding associated protein (GAP) 45, histones and some orthologous proteins from *T.* gondii and *P. falciparum* (Fig. 2; Additional file 6). *Ef*SEV proteins relating to biological processes, cell components and molecular functions are shown in Fig. 3. Also, the protein composition of *Ef*SEV implies the involvement of these proteins in *E. falciformis* sporozoite binding/fusion to the host cell, endocytosis and metabolic processes (Fig. 3). KEGG analysis revealed that *Ef*SEV proteins were enriched in pathways involving endocytosis, protein processing, RNA transport, spliceosome and ribosome, as found in *N. caninum* EV proteins [35], as well as the glutathione metabolism pathway, which is associated with gene expression, DNA and protein synthesis, signal transduction, cytokine production and immune responses (Fig. 3; Additional file 5).

### *Ef*SEVs induced IL-1β, IL-6, IL-17, IL-18 and MCP1 production in MIECs

**Fig. 4:**
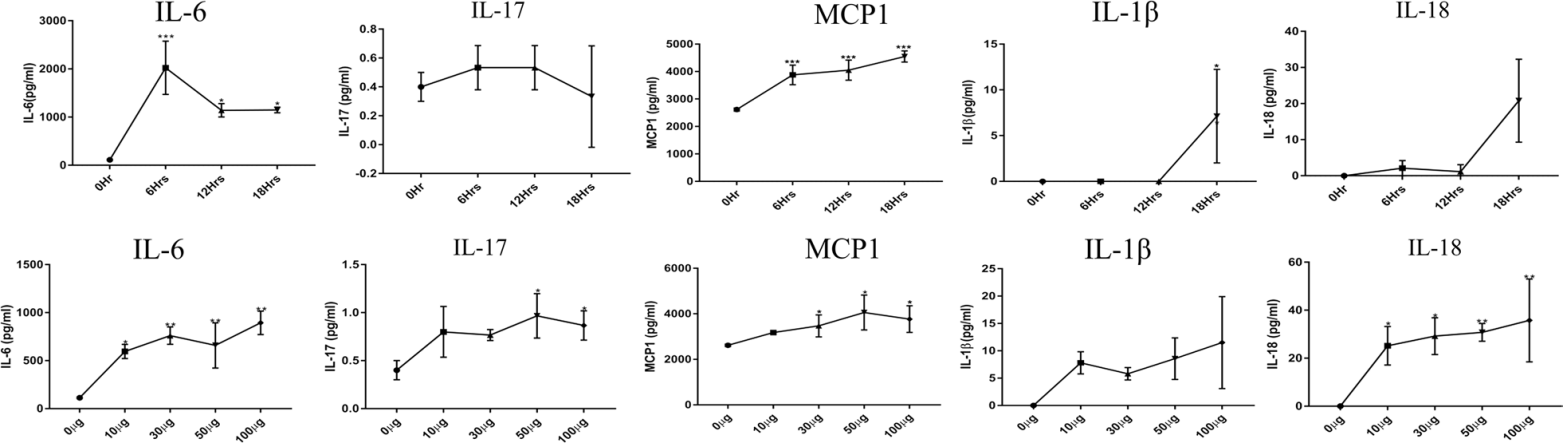
Cytokine and chemokine secretions by MIECs. 1 × 10^6^ MIECs were stimulated with 10, 30, 50 and 100 µg of *Ef*SEVs and PBS (0 µg of *Ef*SEVs) for 24 h. Also, 50 µg of *Ef*SEVs were used to stimulate 1 × 10^6^ MIECs for 6, 12, 18 and 0 h as experimental control. IL-6, IL-17, MCP1, IL-1β and IL-18 secretions in cell culture supernatants were measured by protein microarray kit. The signals of the laser scanning map were extracted with GenePix 6.0 microarray analysis software. Quantitative data obtained from the Quantibody-INF-1Q-Analyzer were analyzed using RayBiotech mouse Inflammation Array 1 software. Cytokine concentrations (pg/ml) were determined by mean fluorescence intensities and linear regression standard curves were generated from the manufacturer’s standard. Each spot on the graph represents quantitative mean ± SD values in triplicate experiments. Asterisks indicate statistical significant differences from the control treatments at: *P < 0.05, **P < 0.001, ***P < 0.0001. Abbreviations: IL, Interleukin; MCPI, monocyte chemoattractant protein 1; MIECs, mouse intestinal epithelial cells; PBS, phosphate-buffered saline

Intestinal epithelial cells are known to secret cytokines and chemokines as well as cellular infiltration during inflammatory responses and parasite-host interactions [17, 42]. Also, *Eimeria* species can induce the secretion of diverse cytokines/chemokines in the host intestinal epithelium, including IL-6, IL-17, IL-18, IL-1β, interferon gamma (INFγ) [17, 44] and MCP1 [43]. However, the reactive antigenicity of *Eimeria*-derived EVs in host inflammatory responses during infection is not fully understood. Therefore, we examined the secretion of MIEC proinflammatory cytokines after *Ef*SEV stimulation. The results showed that MIECs produced quantifiable IL-1β, IL-18, IL-17 and IL-6 in response to *Ef*SEV dose and time stimulations. While the level of IL-6 significantly increased with increasing time of MIEC stimulation by *Ef*SEVs, the production of IL-17 reduced (Fig. 4). In addition, the production of IL-6 and IL-17 increased at high doses of *Ef*SEVs (Fig. 4). Also, the production of MCP1 significantly increased with increasing *Ef*SEV doses and time of stimulation (Fig. 4). The secretion of IL-18 by *Ef*SEVs-stimulated MIECs was initially low but increased with time and with increasing doses of *Ef*SEVs. Similarly, the production of IL-1β only increased significantly at a high dose of *Ef*SEVs (Fig. 4). Taken together, *Ef*SEV dose enhanced the production of proinflammatory cytokines in MIECs whereas the production of MCP1 was significantly increased with increasing time of stimulation and dosage of *Ef*SEVs (Fig. 4).

**Fig. 5:**
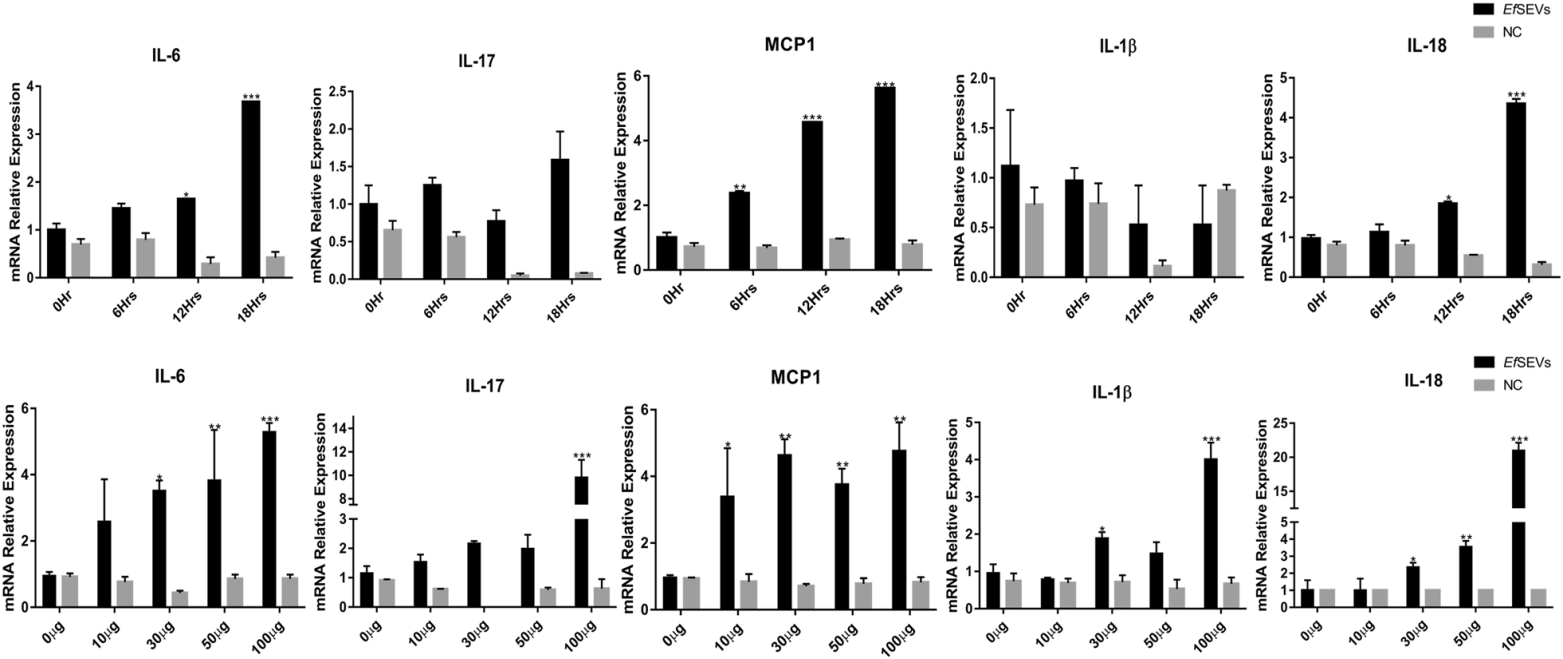
*Eimeria falciformis* sporozoite EVs modified mRNA expressions of IL-6, IL-17, MCP1, IL-1β and IL-18. MIECs (1 × 10^6^) were plated and stimulated with 50 µg of *Ef*SEVs for 6, 12 and 18 h and with 10, 30, 50 and 100 µg of *Ef*SEVs for 24 h. PBS (0 µg of *Ef*SEVs) was used as an experimental control for dose treatments and 0 h was used as control for time-dependent treatments. An equal volume of NC particles was set as negative control. After each time point, RNAs were extracted, and IL-6, IL-17, MCP1, IL-1β and IL-18 expressions were determined by quantitative PCR. Bars represent mean ± standard deviation values in triplicate experiments. Asterisks indicate a statistical significant difference with the experimental control treatment at *P < 0.05, **P < 0.001, ***P < 0.001

**Fig. 6:**
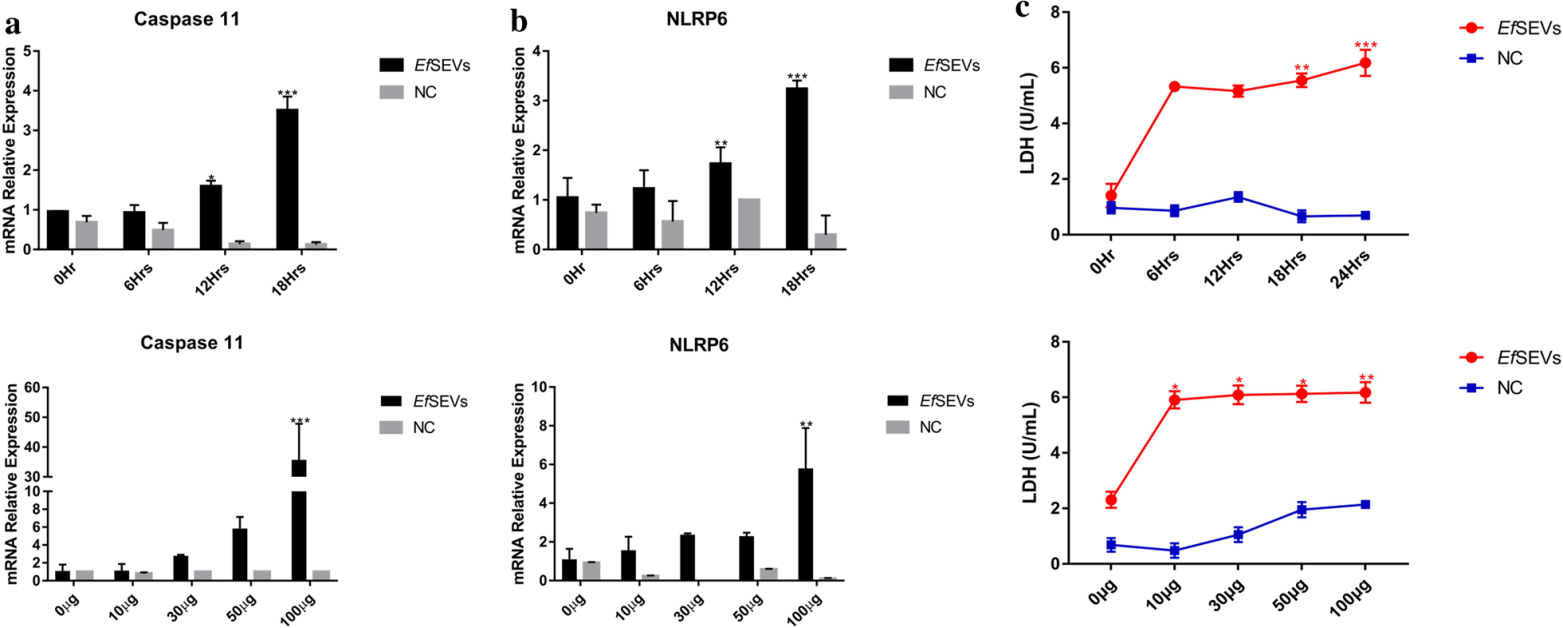
*Eimeria falciformis* sporozoite EVs upregulate caspase 11 and NLRP6 inflammasomes and release LDH. **a**, **b** MIECs (1 × 10^6^) were plated and stimulated with 50 µg of *Ef*SEVs for 6, 12, 18 h and 10, 30, 50 and 100 µg of *Ef*SEVs for 24 h. An equivalent volume of PBS (0 µg of *Ef*SEVs) was used as experimental control for the dose treatment and 0 h was used as control for the time-dependent treatment. An equal volume of NC particles was set as a negative control. RNAs were extracted and analyzed by qPCR for caspase 11 and NLRP6 mRNA expression. Bars represent mean ± standard deviation values in triplicate experiments. The difference in mRNA expression was compared with control treatments and considered to be significant at *P < 0.05, **P < 0.001, ***P < 0.001.** c** *Ef*SEVs induced secretion of LDH in MIECs. 1 × 10^6^ MIECs were stimulated with increasing doses (10, 20, 30, 50, 100 µg) of *Ef*SEVs. Also, 50 µg of *Ef*SEVs was used to stimulate MIECs at increasing duration (0, 6, 12, 18, 24 h). PBS (0 µg of *Ef*SEVs) was used as experimental control for the dose treatment and 0 h as the control for time-dependent treatment. An equal volume of NC particles was set as a negative control in both cases. MIECs were harvested at the end of the treatment and assayed for LDH production using the Chekine™ LDH assay kit in triplicate. Differences in the time- and dose-dependent release of LDH were compared with the control treatments and considered to be significant at *P < 0.05, **P < 0.001, ***P < 0.001. Abbreviations: LDH, Lactate dehydrogenase; NLRP6, nucleotide-oligomerization domain (NOD)-like receptor pyrin 6

Gene expression profiles of the selected cytokines and chemokine also indicated steady and significant upregulation of MCP1 and IL-6 mRNA expression with increasing time of stimulation and *Ef*SEV doses, but the increased IL-17 mRNA expression was just significant at high concentration of *Ef*SEVs (Fig. 5). IL-18 mRNA expression was also significantly upregulated with increasing dose of *Ef*SEVs and time of treatment (Fig. 5). However, mRNA expression of INFγ remained unchanged with increasing time and *Ef*SEV doses (data not shown). In essence, *Ef*SEVs are parasite components that modulate proinflammatory cytokines and chemokine genes during *Eimeria*-host interactions (Fig. 5). The apparent inconsequential change in the expression of the cytokines with NC particles indicated that the observed relative expressions of IL-6, IL-17, IL-18, IL-1β and MCP1 were due to the MIEC responses to *Ef*SEV bioactivities (Fig. 5&6).

### *Ef*SEVs upregulated MIEC pyroptotic inflammasomes and induced the release of LDH

The eventual outcomes of pathogen-host interactions are sometimes marked by inflammation and host cell death [45]. Given the functional association of IL-18 and IL-1β with inflammatory programmed cell death, we investigated the regulatory activities of caspase 11 and NLRP6 in *Ef*SEV-stimulated MIECs by qPCR. The analysis showed that increasing *Ef*SEV dose and increasing duration of stimulation significantly upregulated the mRNA expression of NLRP6 and caspase 11 in MIECs whereas NC particles showed no distinct effects on inflammasome activation (Fig. 6a, b).

Cytosolic protein ligands, such as pathogen-associated molecular patterns (PAMPs), have been shown to activate pyroptosis-dependent IEC death. Specifically, pyroptosis of IECs can occur via the activation of caspase 11 and NLRP6 with a concomitant secretion of IL-18 and IL-1β [46] with the release of cytosolic LDH [39]. Therefore, LDH production by *Ef*SEV-stimulated MIECs was investigated. The increased production of LDH by MIECs was significant from 18 h of *Ef*SEV treatment and with increasing doses of *Ef*SEVs. The result indicated that *Ef*SEVs induced significant production of LDH as a marker for proinflammatory MIEC death. This finding was supported by the observation that NC particles showed no significant involvement in the production of LDH by MIECs (Fig. 6c).

## Discussion

Previous studies have demonstrated that protozoan parasite secretomes include vesicle-bound and VF molecules such as nucleic acids, carbohydrates or glycocnjugates, lipids and proteins [6, 7, 47]. In this study, *E. falciformis* sporozoite EV and VF proteins were resolved from the total secretome formed while the parasite interacted with inactivated MIECs, and the protein components were analyzed by gel electrophoresis and LC–MS/MS. The electrophoretic profiles revealed protein bands in *Ef*SEV and VF gel lanes, but no specific band was visible from NC and NCs, indicating that *Ef*SEVs and VF preparations were free from MIEC secretions (Fig. 1d) (Additional file 5). This observation is in conformity with *T. gondii* tachyzoites which were unable to secret vesicles after being treated with aldehyde [48]. Notwithstanding, *Ef*SEVs are subpopulations of self-assembled spherical and circular-shaped EVs with an expression of Hsp70 as membrane marker (Fig. 1c, d).

The proteomic dataset showed that *Ef*SEVs and the VF fraction comprised membrane-associated and bioactive proteins (Additional file 6). Functionally, the gliding motility of apicomplexan parasites requires actin polymerization [49], and *E. falciformis* sporozoite VF Hsp90 might be involved in host cell invasion, host-parasite interaction, signal transduction and parasite development similarly as found in *Eimeria tenella, T. gondii*, and *P. falciparum* Hsp90 [50]. Also, the Vps51-54 complex has been suggested to mediate host endocytosis of *T. gondii* and *P. falciparum *[51, 52]. Vps54 would possibly play a similar role during *E. falciformis* sporozoite invasion (Fig. 2). Additionally, *Cryptosporidium parvum* EF-1α has been associated with host cell invasion [53], and *E. tenella* protein kinases are immunogens [54] known to inhibit apoptosis and play major roles during stress [55, 56]. Congruently, *E. falciformis* VF proteins are likely to be important for sporozoite motility, survival, invasion and pathogenesis.

Moreover, EF-1α and Hsp70 have been identified as EV markers of *Leishmania major* and other parasitic protozoa [57]. The identification of Hsp70 and EF-1α in *Ef*SEV and VF proteins (Fig. 2) suggests that specific protein could be secreted via two or more secretory pathways during host-parasite interactions. The secretion of Hsp70 by *Eimeria* species is associated with sporozoite formation, pathogenicity, protective immunity and response to stress [5, 58, 59]. Hsp70 has also been reported among EV proteins from *C. parvum,* *N. caninum*, *T. gondii*, *Leishmania* and *Trypanosoma* species [7]. *Ef*SEV proteases are likely to play important roles in *E. falciformis* sporozoite invasion, evasion of immune cells [60], developmental regulation and virulence, as revealed in other Apicomplexa [61], as well as degrading the cell matrix of host tissue [62]. Also, proteases are transcriptional regulators of *E. tenella, Plasmodium* and *T. gondii* life-cycles [5, 63] and, more importantly, the invasion of* Eimeria* sporozoites is accompanied by proteolytic shedding of surface adhesins mediated by protease [64] (Fig. 2).

Refractile body (RB)-derived functional molecules include a number of proteins/enzymes, such as aspartyl proteinase (eimepsin), RB antigen (SO7), and several haloacid dehalogenases such as hydrolase, subtilase, transhydrogenase and ubiquitin, as well as some carbohydrates [65, 66]. Eimepsin is an *Eimeria*-specific antigenic protein [67, 68] that is abundantly secreted in the sporozoite stage [5]. SO7 is also highly immunogenic with conserved antigenic epitopes among *Eimeria* species infecting chicken [69]. SO7 has been reported to effectively stimulate the immune response compared with single fragment vaccine [70], and it also plays important roles in host cell invasion, secretion of microneme proteins and* Eimeria* parasite intracellular survival [69]. In addition, the *E. falciformis* and *E. tenella* leucine aminopeptidase is an enzyme which preferentially catalyzes the hydrolysis of leucine residues at the N-termnus of peptides and proteins [71], which could be crucial for invasion, immune response, parasite excystment [72] and functions similar to the EV-bound aminopeptidase and serine protease of *Acanthoamoeba castellanii* [6, 73]. Identification of *Ef*SEV GAP45 suggests that the *E. falciformis* sporozoite has a trail of EVs during gliding motility [74, 75] as reported in *C. parvum* infection [76]. Also, histones enclosed in EVs secreted by *Leishmania donovani*, *P. falciparum*, *Trypanosoma brucei* and *T. cruzi* could have functions in parasite viability and virulence [77–79], and *Ef*SEV histones could have similar roles. Although *Ef*SEV RNA content was low (data not shown), attending *Ef*SEV histones suggest that *E. falciformis* EV cargo contained nucleic acids. Also, inactivation might have resulted in the loss of MIEC receptors [10] for *E. falciformis* sporozoite attachment, which in turn could have affected the number and composition of proteins in *E. falciformis* sporozoite secretomes.

IL-1β, IL-6, IL-17, IL-18 and MCP1 secretions are known to play crucial roles in *E. tenella, C. parvum* and *T. gondii* infections [80, 81]. IEC IL-18 is constitutive and links innate and adaptive immunity during infection [82]. Similarly, MCP1 recruits monocytes and macrophages and co-regulates IL-1β in inflamed IECs [83]. Therefore, the expression of MCP1 (Figs. 4, 5) indicated that *Ef*SEVs could play critical roles in the transcytosis of monocytes to the site of *E. falciformis* infection [21]. Also, the production of IL-1β could accelerate IL-17 response to PAMPs and stimulate chemokines recruiting leukocytes, T lymphocytes and neutrophils to the intestinal epithelium [84]. Then, *E. falciformis* sporozoites possibly secrete EVs to prevent early infiltration of cytotoxic CD4 + T cells to the site of infection [84] as MIEC IL-1β and IL-17 expressions were low in time-dependent responses to *Ef*SEVs (Figs. 4, 5) and it is comparable  to the expressions of the IL-17 subset and neutrophils in *T. cruzi*-infected mice [85]. However, at a later time and high concentrations, *Ef*SEVs induced significant expression of IL-17, as reported in mice infected with *Leishmania major* [86], suggesting that *Ef*SEVs are potent, dose-dependent activators of inflammation and that the observed latent inflammatory effect of *Ef*SEVs on MIEC might be associated with *E. falciformis* sporozoite behavior for intracellular survival.

It has been previously reported that *Trichomonas vaginalis*, *Leishmania infantum*, and *P. falciparum* EVs induced secretion of IL-6 from immune cells [7]. However, the secretion of IL-6 could be related to anti-inflammatory and anti-apoptotic responses in IECs [87, 88]. It is possible that *E. falciformis* sporozoites secrete EVs to suppress MIEC apoptotic activities because it has been suggested that *T. vaginalis* exosomes upregulate IL-6 to suppress other proinflammatory cytokines [89]. This is in contrast to extracellular acidosis that has been reported to transiently modulate IL-6 expression in rat epithelial cell [90]. Nevertheless, *Ef*SEVs enclosed potential danger/damage/stress-associated ligands that activated the release of endogenous IL-18 and IL-1β as proinflammatory cytokines associated with inflammasome complexes [91] (Figs. 5, 6). Also, *Ef*SEVs [35, 41] were able to bind green fluorescent dye and aggregate around the MIEC membrane with some degree of internalization at the later experimental time, as well as involvemnet  in MIEC membrane disruption or sphericalization (Additional file 5). The cytoplasmic incorporation of *Ef*SEVs by MIECs might render them as cytosolic ligands that subsequently activate inflammasome complexes [92].

Inflammasomes are vital innate immune complexes in protozoa-host interactions [93] and are activated by intracellular pattern-recognition receptors (or sensors) that recognize pathogen- danger- [94] and stress-associated ligands [95]. Inflammasomes include NLRPs, AIM2 and caspases [93, 96]. NLRP inflammasome families contribute to intestinal disease pathogenesis while caspases participate in proinflammatory processes and pathophysiology of enteric pathogens [20, 97, 98]. In the present study, upregulation of MIEC caspase 11 and NLRP6, as well as dose-dependent upregulation of IL-18 and IL-1β (Figs. 5, 6) are comparable with inflammatory cascades and NLRP6 activation during mice colonic inflammation and tumorigenesis [46, 96]. Likewise, the activation of NLRP6 during *Cryptosporidium tyzzeri* infection in mice caused a luminal release of IL-18 [99, 100]. It is therefore not unlikely that *C. tyzzeri*-derived EVs, though not characterized, might have played pivotal role in the activation of NLRP6 in *C. tyzzeri*-infected mice, as observed in this study (Fig. 6a, b). Mechanistically, non-canonical caspase 11 is activated when toxins (e.g. bacterial lipopolysaccharide [LPS]) find their way into the host cell cytoplasm [97, 101]. In the absence of the bacteria, *Brucella melitensis* LPS induced caspase 11 in murine enterocytes [102] like *Ef*SEVs in this study. Moreover, the production of IL-1β, IL-18 and caspase 11 have been posited as markers for intestinal inflammation in response to pathogens [92, 103].

Additionally, caspase 11-dependent pyroptosis, in association with IL-18, is a particularly important host immune mechanism against intracellular pathogens [92, 102]. Also, the production of cytosolic LDH has been defined as a marker for inflammasome-dependent pyroptosis [98, 104, 105]. Upregulation of caspase 11 and NLRP6 with a significant release of LDH as well as dose- and time-dependent secretion of IL-1β and IL-18 [106] signified that *Ef*SEVs are potential activators of pyroptosis in MIECs (Figs. 5, 6). Comparatively, the MIEC inflammatory response and the release of the cell death signal induced by *Ef*SEVs were synchronous with the time of *E. falciformis* sporozoite intracellular egress [30]. However, the mechanism by which intracellular parasites secrete a regulated amount of EVs to temporarily delay host responses until the completion of intracellular development requires further investigation. It is therefore possible that inflammatory responses and host cell death during *Eimeria* infection are not entirely passive due to intraepithelial distress, but are prefigured by *Eimeria* parasite behavior for survival and successful completion of the life-cycle.

## Conclusion

This study emphasized host-parasite interaction as an additional mechanism by which the protozoan parasite *E. falciformis* secretes EVs and VF molecules. The study unveils the protein compositions of *E. falciformis* sporozoite secretome as well as the MIEC inflammatory response to *E. falciformis* sporozoite EVs. Specifically, *E. falciformis* sporozoite EVs contained important antigenic and immunogenic proteins as well as other apicomplexan proteins that are implicated in motility, invasion, pathogenesis, survival and host cell degradation. Also, *E. falciformis* VF proteins encompassed proteins involved in pathogenesis, motility and vesicle formation. Furthermore, *E. falciformis* sporozoite EVs induced the release of proinflammatory molecules such as IL-6, IL-17, IL-1β and IL-18 as well as the upregulation of pyroptosis-dependent caspase 11 and NLRP6, at the least, in a dose-dependent manner. Essentially, *E. falciformis* EVs are components of danger-associated molecular patterns (DAMPs) with protein- and gene-regulatory activities that support pyroptosis-dependent response. Unlike parasite protein characterization through extraneous stimuli, the model of the study could be adapted to ascertain secretory motifs and constitutive antigens/immunogens in *Eimeria* species for the development of vaccines against poultry coccidiosis. Also, it will benefit our understanding to further describe the classical components of NLRP6- and caspase 11-mediated pyroptosis during *E. falciformis* infection.

## Supplementary Information

**Additional file 1: **Non-treated control before incubation.

**Additional file 2: **Parasite and inactivated MIECs before incubation.

**Additional file 3: **Non-treated control after incubation.

**Additional file 4: **Parasite and inactivated MIECs after incubation.

**Additional file 5: **Electrophoretic gel, bioinformatic result, and result from non-treated control.

**Additional file 6: **Mass spectrometry data set for identified proteins.

## Abbreviations

AIM2: Absent in melanoma 2
ECL: Enhanced chemiluminescence
*Ef*SEVs: *E. falciformis* sporozoite EVs
EVs: Extracellular vesicles
IL: Interleukin
LC–MS/MS: Liquid chromatography–tandem mass spectrometry
LPS: Lipopolysaccharide
MCP1: C–C motif chemokine 2/monocyte chemoattractant protein 1
MIECs: Mouse intestinal epithelial cells
NLRP6: Nucleotide-oligomerization domain (NOD)-like receptor pyrin 6
PAMP: Pathogen-associated molecular pattern
PBS: Phosphate-buffered saline
ODG: OptiPrep™ density gradient
SDS-PAGE: Sodium dodecyl sulphate–polyacrylamide gel electrophoresis
VF: Vesicle-free

## References

[1] Montaner S, Galiano A, Trelis M, Martin-jaular L, Portillo HA, Bernal D (2014). The role of extracellular vesicles in modulating the host immune response. Front Immunol.

[2] Marcilla A, Trelis M, Cortés A, Sotillo J, Cantalapiedra F, Minguez MT (2012). Extracellular vesicles from parasitic helminths contain specific excretory/secretory proteins and are internalized in intestinal host cells. PLoS ONE.

[3] Pope SM, Lässer C, Lasser C (2013). *Toxoplasma gondii* infection of fibroblasts causes the production of exosome-like vesicles containing a unique array of mRNA and miRNA transcripts compared to serum starvation. J Extracell Vesicles.

[4] Graewe S, Rankin KE, Lehmann C, Deschermeier C, Hecht L, Froehlke U (2011). Hostile takeover by *Plasmodium*: reorganization of parasite and host cell membranes during liver stage egress. PLoS Pathog.

[5] Olajide JS, Qu Z, Yang S, Oyelade OJ, Cai J (2022). Eimeria proteins: order amidst disorder. Parasit Vectors.

[6] Gonçalves DDS, Ferreira S, Liedke SC, Gomes KX, De OGA, Lopes PE (2018). Extracellular vesicles and vesicle-free secretome of the protozoa *Acanthamoeba castellanii* under homeostasis and nutritional stress and their damaging potential to host cells. Virulence.

[7] Olajide JS, Cai J (2020). Perils and promises of pathogenic protozoan extracellular vesicles. Front Cell Infect Microbiol.

[8] Castelli G, Bruno F, Saieva L, Alessandro R, Galluzzi L, Diotallevi A (2019). Exosome secretion by *Leishmania infantum* modulate the chemotactic behavior and cytokinic expression creating an environment permissive for early infection. Exp Parasitol..

[9] Heitlinger E, Spork S, Lucius R, Dieterich C (2014). The genome of *Eimeria falciformis*—reduction and specialization in a single host apicomplexan parasite. BMC Genomics.

[10] Li W, Wang M, Chen Y, Chen C, Liu X, Sun X (2020). EtMIC3 and its receptors BAG1 and ENDOUL are essential for site-specific invasion of *Eimeria tenella* in chickens. Vet Res.

[11] Deolindo P, Evans-Osses I, Ramirez MI (2013). Microvesicles and exosomes as vehicles between protozoan and host cell communication. Biochem Soc Trans.

[12] Szempruch AJ, Dennison L, Kieft R, Harrington JM, Hajduk SL (2016). Sending a message: extracellular vesicles of pathogenic protozoan parasites. Nat Rev Microbiol.

[13] Silva VO, Maia MM, Torrecilhas AC, Taniwaki NN, Namiyama GM, Oliveira KC (2018). Extracellular vesicles isolated from *Toxoplasma*
*gondii* induce host immune response. Parasite Immunol.

[14] Wu Z, Wang L, Li J, Wang L, Wu Z, Sun X (2018). Extracellular vesicle-mediated communication within host-parasite interactions. Front Immunol.

[15] Valadi H, Ekström K, Bossios A, Sjöstrand M, Lee JJ, Lötvall JO (2007). Exosome-mediated transfer of mRNAs and microRNAs is a novel mechanism of genetic exchange between cells. Nat Cell Biol.

[16] Huang G, Zhang S, Zhou C, Tang X, Li C, Wang C (2018). Influence of *Eimeria falciformis* infection on gut microbiota and metabolic pathways in mice. Infect Immun.

[17] Schmid M, Heitlinger E, Spork S, Mollenkopf HJ, Lucius R, Gupta N (2014). *Eimeria falciformis* infection of the mouse caecum identifies opposing roles of IFNγ-regulated host pathways for the parasite development. Mucosal Immunol.

[18] Ehret T, Spork S, Dieterich C, Lucius R, Heitlinger E (2017). Dual RNA-seq reveals no plastic transcriptional response of the coccidian parasite *Eimeria falciformis* to host immune defenses. BMC Genomics.

[19] Straub KW, Cheng SJ, Sohn CS, Bradley PJ (2009). Novel components of the Apicomplexan moving junction reveal conserved and coccidia-restricted elements. Cell Microbiol.

[20] Fink SL, Cookson BT (2005). Apoptosis, pyroptosis, and necrosis: mechanistic description of dead and dying eukaryotic cells. Infect Immun.

[21] Al Klifeh E, Balard A, Jarquín-Diaz VH, Weyrich A, Wibbelt G, Heitlinger E (2019). Eimeria falciformis BayerHaberkorn1970 and novel wild derived isolates from house mice : differences in parasite lifecycle, pathogenicity and host immune reactions. bioRxiv.

[22] Pogonka T, Schelzke K, Stange J, Papadakis K, Steinfelder S, Liesenfeld O (2010). CD8+ cells protect mice against reinfection with the intestinal parasite *Eimeria*
*falciformis*. Microbes Infect.

[23] Suresh P, Rehg JE (1996). Comparative evaluation of several techniques for purification of *Cryptosporidium*
*parvum* oocysts from rat feces. J Clin Microbiol.

[24] Cai X, Lorraine Fuller A, McDougald LR, Tan X, Cai J, Wang F (2007). Biochemical characterization of enoyl reductase involved in Type II fatty acid synthesis in the intestinal coccidium *Eimeria tenella* (Phylum Apicomplexa). FEMS Microbiol Lett.

[25] El-ashram S, Suo X (2017). Electrical cream separator coupled with vacuum filtration for the purification of eimerian oocysts and trichostrongylid eggs. Sci Rep.

[26] Iwamoto T, Yamada K, Shimizu M, Totsuka M (2011). Establishment of intestinal epithelial cell lines from adult mouse small and large intestinal crypts. Biosci Biotechnol Biochem.

[27] Gvan N, Raposo A, Candalh C, Boussac M, Hershberg R, Cerf-Bensussan N (2001). Intestinal epithelial cells secrete exosome-like vesicles. Gastroenterology.

[28] Pérez-Cabezas B, Santarém N, Cecílio P, Silva C, Silvestre R, Catita J (2018). More than just exosomes: distinct *Leishmania infantum* extracellular products potentiate the establishment of infection. J Extracell Vesicles.

[29] Lutz K, Taubert A, Zahner H, Menge C, Hermosilla C, Stamm I (2008). Fluorescent *Eimeria*
*bovis* sporozoites and meront stages in vitro: a helpful tool to study parasite–host cell interactions. Parasitol Res.

[30] Mesfin GM, Bellamy JEC (1978). The life cycle of *Eimeria*
*falciformis* var. Pragensis (Sporozoa: Coccidia) in the mouse, Mus musculus. J Parasitol.

[31] Ribeiro KS, Vasconcellos CI, Soares RP, Mendes MT, Ellis CC, Aguilera-Flores M (2018). Proteomic analysis reveals different composition of extracellular vesicles released by two *Trypanosoma*
*cruzi* strains associated with their distinct interaction with host cells. J Extracell Vesicles.

[32] Tauro BJ, Mathias RA, Greening DW, Gopal SK, Ji H, Kapp EA (2013). Oncogenic H-Ras reprograms madin-darby canine kidney (MDCK) cell-derived exosomal proteins following epithelial-mesenchymal transition. Mol Cell Proteomics.

[33] Caeiro LD, Alba-Soto CD, Rizzi M, Solana ME, Rodriguez G, Chidichimo AM (2018). The protein family TcTASV-C is a novel *Trypanosoma cruzi* virulence factor secreted in extracellular vesicles by trypomastigotes and highly expressed in bloodstream forms. PLoS Negl Trop Dis.

[34] Li Y, Xiu F, Mou Z, Xue Z, Du H, Zhou C (2018). Exosomes derived from *Toxoplasma gondii* stimulate an inflammatory response through JNK signaling pathway. Nanomedicine.

[35] Li S, Gong P, Tai L, Li X, Wang X, Zhao C (2018). Extracellular vesicles secreted by *Neospora caninum* are recognized by toll-like receptor 2 and modulate host cell innate immunity through the MAPK signaling pathway. Front Immunol.

[36] Wowk PF, Zardo ML, Miot HT, Goldenberg S, Carvalho PC, Mörking PA (2017). Proteomic profiling of extracellular vesicles secreted from *Toxoplasma gondii*. Proteomics.

[37] Huang T, He J (2017). Characterization of extracellular vesicles by size-exclusion high-performance liquid chromatography (HPLC). Methods Mol Biol.

[38] Wolff BS, Alshawi SA, Feng LR, Juneau PL, Saligan LN (2021). Inflammation plays a causal role in fatigue-like behavior induced by pelvic irradiation in mice. Brain Behav Immun Health.

[39] den Hartigh AB, Fink SL (2018). Pyroptosis Induction and detection. Curr Protoc Immunol.

[40] Rayamajhi M, Zhang Y, Miao E (2013). Detection of pyroptosis by measuring released lactate dehydrogenase activity. Methods Mol Biol.

[41] Li Y, Liu Y, Fangming X, Jianing W, Cong H, He S (2018). Characterization of exosomes derived from *Toxoplasma gondii* and their functions in modulating immune responses. Int J Nanomed.

[42] Stadnyk AW (2002). Intestinal epithelial cells as a source of inflammatory cytokines and chemokines. Can J Gastroenterol.

[43] Hong YH, Lillehoj HS, Lee SH, Dalloul RA, Lillehoj EP (2006). Analysis of chicken cytokine and chemokine gene expression following *Eimeria acervulina* and *Eimeria tenella* infections. Vet Immunol Immunopathol.

[44] Hong YH, Lillehoj HS, Lillehoj EP, Lee SH (2006). Changes in immune-related gene expression and intestinal lymphocyte subpopulations following *Eimeria maxima* infection of chickens. Vet Immunol Immunopathol.

[45] Malireddi RKS, Kanneganti TD (2013). Role of type I interferons in inflammasome activation, cell death, and disease during microbial infection. Front Cell Infect Microbiol.

[46] Ghimire L, Paudel S, Jin L, Jeyaseelan S (2020). The NLRP6 inflammasome in health and disease. Mucosal Immunol.

[47] Nievas YR, Coceres VM, Midlej V, de Souza W, Benchimol M, Pereira-Neves A (2018). Membrane-shed vesicles from the parasite *Trichomonas vaginalis*: characterization and their association with cell interaction. Cell Mol Life Sci.

[48] Ramírez-Flores CJ, Cruz-Mirón R, Mondragón-Castelán ME, González-Pozos S, Ríos-Castro E, Mondragón-Flores R (2019). Proteomic and structural characterization of self-assembled vesicles from excretion/secretion products of *Toxoplasma gondii*. J Proteomics.

[49] Marq J, Stratmann R, Limenitakis J (2010). Article functional dissection of the apicomplexan glideosome molecular architecture. Cell Host Microbe.

[50] Péroval M, Péry P, Labbé M (2006). The heat shock protein 90 of *Eimeria tenella* is essential for invasion of host cell and schizont growth. Int J Parasitol.

[51] Jimenez-ruiz E, Morlon-guyot J, Daher W, Meissner M (2016). Vacuolar protein sorting mechanisms in apicomplexan parasites. Mol Biochem Parasitol.

[52] Spielmann T, Gras S, Sabitzki R, Meissner M (2020). Endocytosis in *Plasmodium* and *Toxoplasma* parasites. Trends Parasitol.

[53] Matsubayashi M, Teramoto-kimata I, Uni S, Lillehoj HS, Matsuda H (2013). Elongation Factor-1 is a novel protein associated with host cell invasion and a potential protective antigen of *Cryptosporidium parvum*. J Biol Chem.

[54] Zhang Z, Wang S, Li C, Liu L (2017). Immunoproteomic analysis of the protein repertoire of unsporulated *Eimeria*
*tenella* oocysts. Parasite.

[55] Diallo MA, Sausset A, Gnahoui-David A, Silva ARE, Brionne A, Le Vern Y (2019). *Eimeria tenella* rop kinase etrop1 induces g0/g1 cell cycle arrest and inhibits host cell apoptosis. Cell Microbiol.

[56] Sullivan WJ, Narasimhan J, Bhatti MM, Wek RC (2004). Parasite-specific eIF2 ( eukaryotic initiation factor-2) kinase required for stress-induced translation control. Biochem J.

[57] de Souza W, Barrias ES (2020). Membrane-bound extracellular vesicles secreted by parasitic protozoa: cellular structures involved in the communication between cells. Parasitol Res Parasitol Res.

[58] del Cacho E, Gallego M, López-Bernad F, Quílez J, Sánchez-Acedo C (2005). Differences in Hsp70 expression in the sporozoites of the original strain and precocious lines of *Eimeria tenella*. J Parasitol.

[59] Bogado ALG, Martins GF, Sasse JP, Guimarães JDS, Garcia JL (2017). Molecular cloning, sequencing, and expression of *Eimeria tenella* HSP70 partial gene. Genet Mol Res.

[60] Benns HJ, Tate EW, Child MA, Tate HJ (2018). Activity-based protein profiling for the study of parasite biology. Curr Top Microbiol Immunol.

[61] Liu R, Ma X, Liu A, Zhang L, Cai J, Wang M (2014). Identification and characterization of a cathepsin-L-like peptidase in *Eimeria tenella*. Parasitol Res.

[62] Pi˜na-Vazquez C, Reyes-Lopez M, Ortız-Estrada G, de la Garza M, Serrano-Luna J (2012). Host-parasite interaction : parasite-derived and -induced proteases that degrade human extracellular matrix. J Parasitol Res.

[63] Matsubayashi M, Kawahara F, Hatta T, Yamagishi J, Miyoshi T, Anisuzzaman (2016). Transcriptional profiles of virulent and precocious strains of Eimeria tenella at sporozoite stage, novel biological insight into attenuated asexual development. Infect Genet Evol..

[64] Zheng J, Gong P, Jia H, Li M, Zhang G, Zhang X (2014). *Eimeria tenella* rhomboid 3 has a potential role in microneme protein cleavage. Vet Parasitol.

[65] de Venevelles P, François Chich J, Faigle W, Lombard B, Loew D, Péry P (2006). Study of proteins associated with the *Eimeria tenella* refractile body by a proteomic approach. Int J Parasitol.

[66] Vermeulen AN, Kok JJ, Van den Boogaart P, Dijkema R, Claessens JA (1993). Eimeria refractile body proteins contain two potentially functional characteristics : transhydrogenase and carbohydrate transport. FEMS Microbiol Lett.

[67] Jean L, Grosclaude J, Labbé M, Tomley F, Péry P (2000). Differential localisation of an *Eimeria tenella* aspartyl proteinase during the infection process. Int J Parasitol.

[68] De Venevelles P, Chich JF, Faigle W, Loew D, Labbé M, Girard-Misguich F (2004). Towards a reference map of *Eimeria tenella* sporozoite proteins by two-dimensional electrophoresis and mass spectrometry. Int J Parasitol.

[69] Rafiqi SI, Garg R, Reena KK, Ram H, Singh M, Banerjee PS (2018). Immune response and protective efficacy of *Eimeria tenella* recombinant refractile body protein, EtSO7, in chickens. Vet Parasitol.

[70] Song X, Xu L, Yan R, Huang X, Li X (2015). Construction of *Eimeria*
*tenella* multi-epitope DNA vaccines and their protective efficacies against experimental infection. Vet Immunol Immunopathol.

[71] Li JG, Gu WY, Tao JP, Liu ZP (2009). The effects of S-nitroso-glutathione on the activities of some isoenzymes in *Eimeria*
*tenella* oocysts. Vet Parasitol.

[72] Fetterer RH, Miska KB, Barfield RC (2006). Partial purification and characterization of an aminopeptidase from *Eimeria tenella*. J Parasitol.

[73] Lin WC, Tsai CY, Huang JM, Wu SR, Chu LJ (2019). Quantitative proteomic analysis and functional characterization of *Acanthamoeba castellanii* exosome—like vesicles. Parasit Vectors.

[74] Roditi I (2016). The languages of parasite communication. Mol Biochem Parasitol.

[75] Sloves P, Morelle W, Alayi TD, Slomianny C, Werkmeister E, Schaeffer C (2011). Unusual N -glycan structures required for trafficking *Toxoplasma gondii* GAP50 to the inner membrane complex regulate host cell entry through parasite motility. Mol Cell Proteomics.

[76] Gong AY, Hu G, Zhou R, Liu J, Feng Y, Soukup GA (2011). MicroRNA-221 controls expression of intercellular adhesion molecule-1 in epithelial cells in response to *Cryptosporidium parvum* infection. Int J Parasitol.

[77] Silverman JM, Clos J, Horakova E, Wang AY, Wiesgigl M, Kelly I (2010). *Leishmania* exosomes modulate innate and adaptive immune responses through effects on monocytes and dendritic cells. J Immunol.

[78] Sisquella X, Regev-Rudzki N, Gerlic M, Schofield L, Sampaio NG, Hansen DS (2017). Malaria parasite DNA-harbouring vesicles activate cytosolic immune sensors. Nat Commun.

[79] Sullivan WJ, Naguleswaran A, Angel SO (2006). Histones and histone modifications in protozoan parasites. Cell Microbiol.

[80] Buzoni-Gatel D, Schulthess J, Menard LC, Kasper LH (2006). Mucosal defences against orally acquired protozoan parasites, emphasis on *Toxoplasma*
*gondii* infections. Cell Microbiol.

[81] Zhang L, Liu R, Song M, Hu Y, Pan B, Cai J (2013). *Eimeria tenella*: Interleukin 17 contributes to host immunopathology in the gut during experimental infection. Exp Parasitol.

[82] van de Veerdonk FL, Netea MG, Dinarello CA, Joosten LAB (2011). Inflammasome activation and IL-1β and IL-18 processing during infection. Trends Immunol.

[83] Reinecker HC, Loh EY, Ringler DJ, Mehta A, Rombeau JL, MacDermott RP (1995). Monocyte-chemoattractant protein 1 gene expression in intestinal epithelial cells and inflammatory bowel disease mucosa. Gastroenterology.

[84] Ge Y, Huang M, Yao YM (2020). Biology of Interleukin-17 and its pathophysiological significance in sepsis. Front Immunol.

[85] Erdmann H, Roßnagel C, Böhme J, Iwakura Y, Jacobs T, Schaible UE (2013). IL-17A promotes macrophage effector mechanisms against *Trypanosoma cruzi* by trapping parasites in the endolysosomal compartment. Immunobiology.

[86] Gonzalez-Lombana C, Gimblet C, Bacellar O, Oliveira WW, Passos S, Carvalho LP (2013). IL-17 mediates immunopathology in the absence of il-10 following *Leishmania major* infection. PLoS Pathog.

[87] Chen L, Deng H, Cui H, Fang J, Zuo Z, Deng J (2018). Inflammatory responses and inflammation-associated diseases in organs. Oncotarget.

[88] Hu B, Elinav E, Huber S, Strowig T, Hao L, Hafemann A (2013). Microbiota-induced activation of epithelial IL-6 signaling links inflammasome-driven inflammation. Proc Natl Acad Sci USA.

[89] Twu O, De MN, Lustig G, Stevens GC, Vashisht AA, Wohlschlegel JA (2013). *Trichomonas vaginalis* exosomes deliver cargo to host cells and mediate host : parasite interactions. PLoS Pathog.

[90] Riemann A, Reime S, Gießelmann M, Thews O (2020). Extracellular acidosis regulates the expression of inflammatory mediators in rat epithelial cells. Adv Exp Med Biol.

[91] Redpath SA, Fonseca NM, Perona-Wright G (2014). Protection and pathology during parasite infection: IL-10 strikes the balance. Parasite Immunol.

[92] Lei-Leston AC, Murphy AG, Maloy KJ (2017). Epithelial cell inflammasomes in intestinal immunity and inflammation. Front Immunol.

[93] de Carvalho RVH, Zamboni DS (2020). Inflammasome activation in response to intracellular protozoan parasites. Trends Parasitol.

[94] Man SM (2018). Inflammasomes in the gastrointestinal tract: infection, cancer and gut microbiota homeostasis. Nat Rev Gastroenterol Hepatol.

[95] Gagliani N, Palm NW, de Zoete MR, Flavell RA (2014). Inflammasomes and intestinal homeostasis: regulating and connecting infection, inflammation and the microbiota. Int Immunol.

[96] Chen GY, Liu M, Wang F, Bertin J, Núñez G (2011). A Functional Role for Nlrp6 in intestinal inflammation and tumorigenesis. J Immunol.

[97] de Gassart A, Martinon F (2015). Pyroptosis: caspase-11 unlocks the gates of death. Immunity.

[98] Yin J, Sheng B, Yang K, Sun L, Xiao W, Yang H (2019). The protective roles of NLRP6 in intestinal epithelial cells. Cell Prolif.

[99] Placea DE, Kanneganti T-D (2017). Recent advances in inflammasome biology. Physiol Behav.

[100] Sateriale A, Gullicksrud JA, Engiles JB, McLeod BI, Kugler EM, Henao-Mejia J (2021). The intestinal parasite *Cryptosporidium* is controlled by an enterocyte intrinsic inflammasome that depends on NLRP6. Proc Natl Acad Sci USA.

[101] Zamboni DS, Sacks DL (2019). Inflammasomes and *Leishmania*: in good times or bad, in sickness or in health. Curr Opin Microbiol.

[102] Lacey CA, Mitchell WJ, Dadelahi AS (2018). caspase-1 and caspase-11 mediate pyroptosis, inflammation, and control of *Brucella* joint infection. Infect Immun.

[103] Lopez-Castejon G, Brough D (2011). Understanding the mechanism of IL-1β secretion. Cytokine Growth Factor Rev.

[104] Winsor N, Krustev C, Bruce J, Philpott DJ, Girardin SE (2019). Canonical and noncanonical inflammasomes in intestinal epithelial cells. Cell Microbiol.

[105] Huang X, Feng Y, Xiong G, Whyte S, Duan J, Yang Y (2019). Caspase-11, a specific sensor for intracellular lipopolysaccharide recognition, mediates the non-canonical inflammatory pathway of pyroptosis. Cell Biosci.

[106] Frank D, Vince JE (2019). Pyroptosis versus necroptosis: similarities, differences, and crosstalk. Cell Death Differ.

