# Supplementary material for: Eimeria falciformis secretes extracellular vesicles to modulate proinflammatory response during interaction with mouse intestinal epithelial cells
Source: Parasit Vectors. 2022 Jul 8;15:245. doi: 10.1186/s13071-022-05364-x (PMC9270845; doi:10.1186/s13071-022-05364-x)
Supplement: Supplementary file 5 — Additional file 5: Electrophoretic gel, bioinformatic result, and result from non-treated control. [file 13071_2022_5364_MOESM5_ESM.pdf]

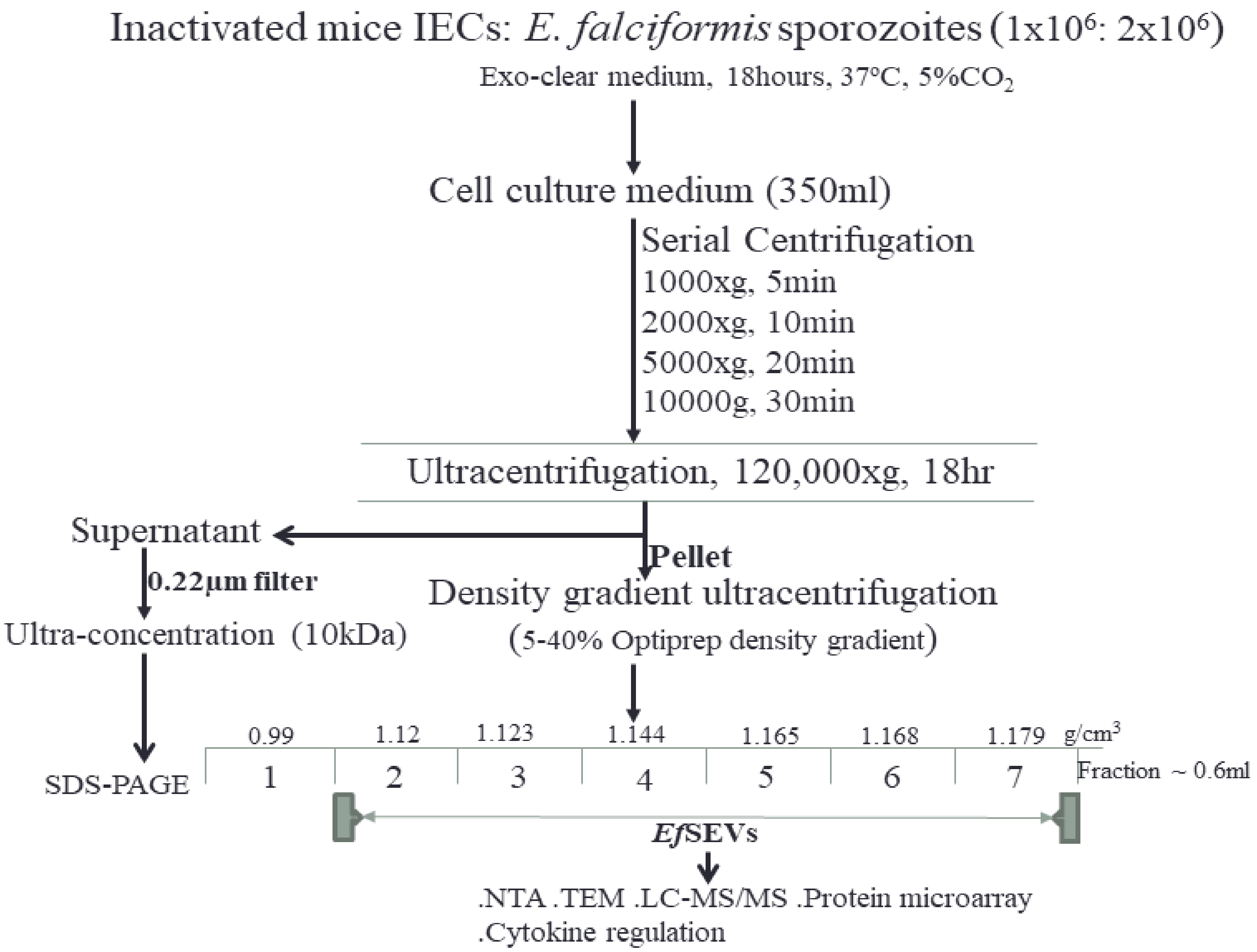
Inactivated mice IECs: E. falciformis sporozoites (1x10^6: 2x10^6)
Exo-clear medium, 18hours, 37°C, 5%CO2
Cell culture medium (350ml)
Serial Centrifugation
1000xg, 5min
2000xg, 10min
5000xg, 20min
10000g, 30min
Ultracentrifugation, 120,000xg, 18hr
Supernatant
0.22μm filter
Ultra-concentration (10kDa)
SDS-PAGE
Pellet
Density gradient ultracentrifugation
(5-40% Optiprep density gradient)
0.99
1.12
1.123
1.144
1.165
1.168
1.179
g/cm3
1
2
3
4
5
6
7
Fraction ~ 0.6ml
EfSEVs
.NTA .TEM .LC-MS/MS .Protein microarray
.Cytokine regulation

a

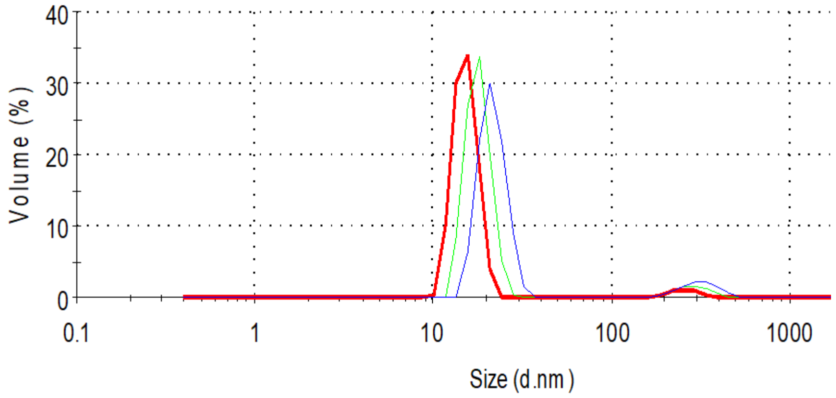

NTA pictogram of particles derived from non-treatment control experiment (NC). Size 9.70-43.36nm with density between 0.57-1.104gcm^{-3}

b

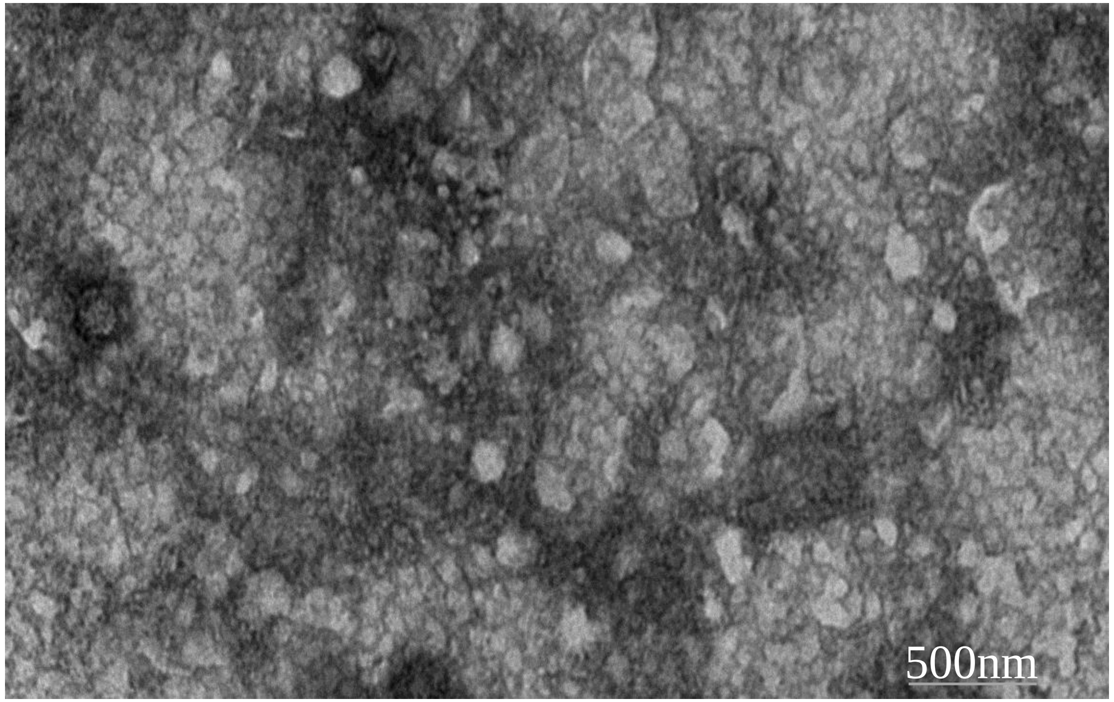

TEM image of particles in non-treatment control experiment (NC)

Figure 2-2: (a) NTA measurement of the NC particles (b) TEM image of NC particles

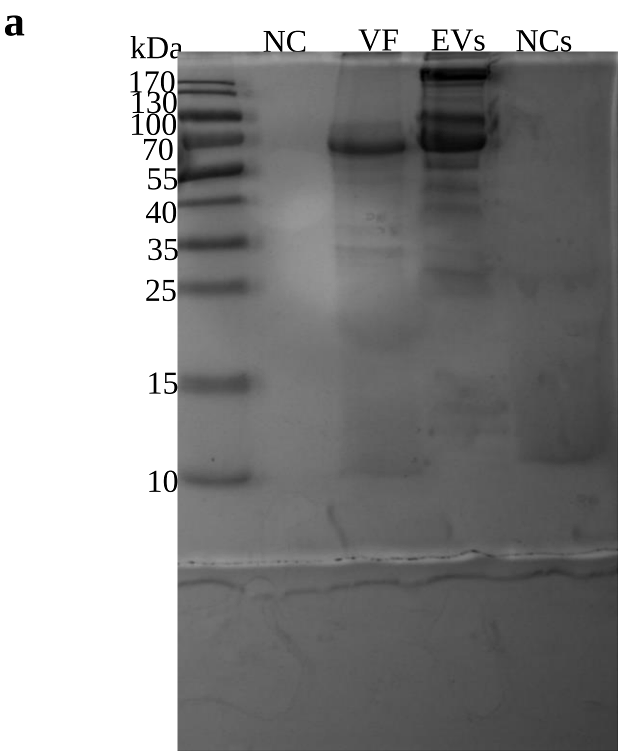

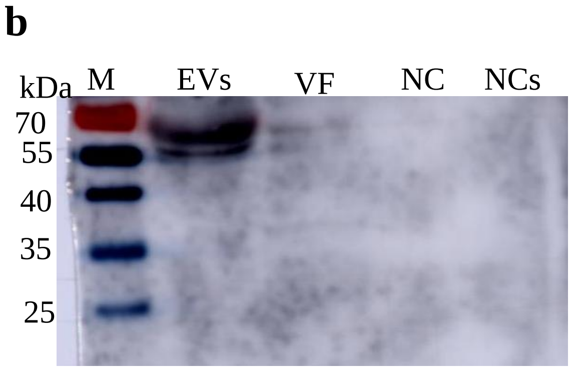

KEY:
NC: ODG particles from NC
NCs: supernatant from NC
VF: vesicles free proteins from parasite culture supernatant
EVs: EVs pellets from parasite culture

Figure 2-3: () SDS-PAGE gels from parasite culture and control experiment (B) Western blot PVDF membrane

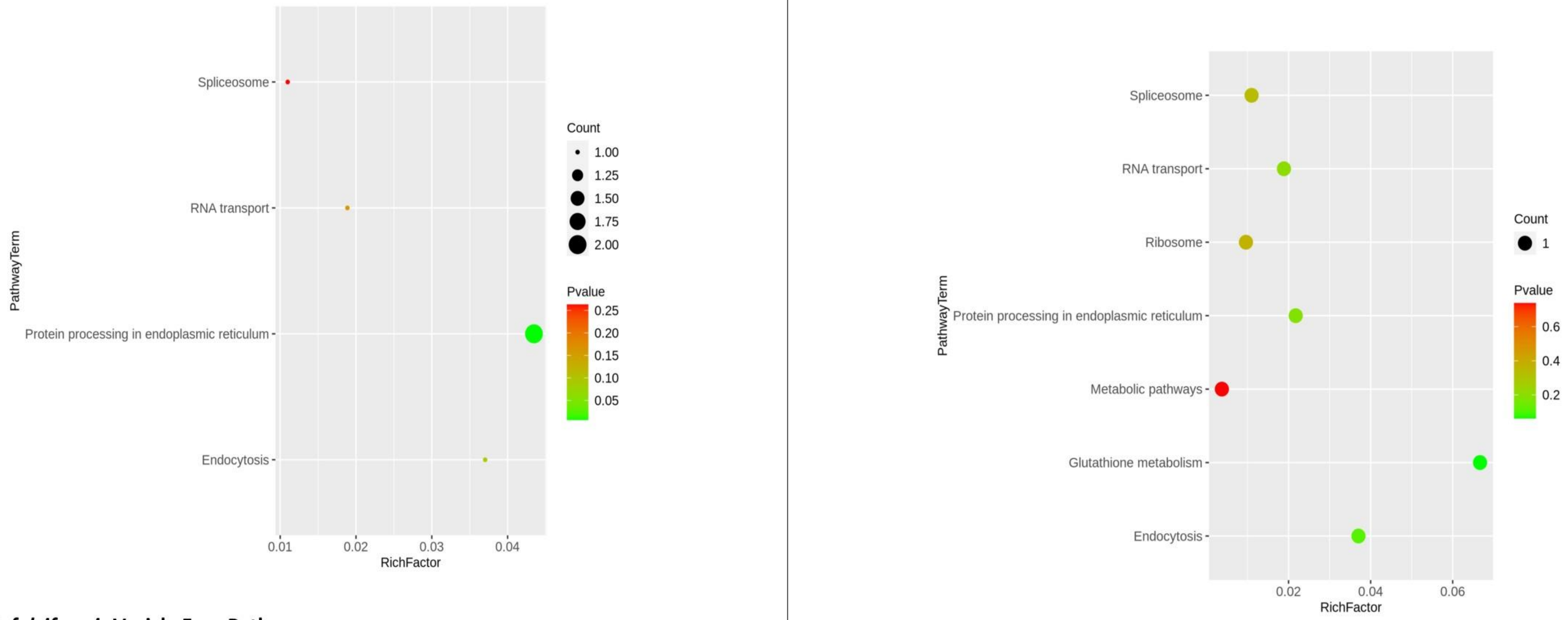

E. falciformis **Vesicle Free Pathway**

Ef**EVs pathway**

Figure 2-4: KEGG pathway for identified proteins from EVs and vesicle free proteins

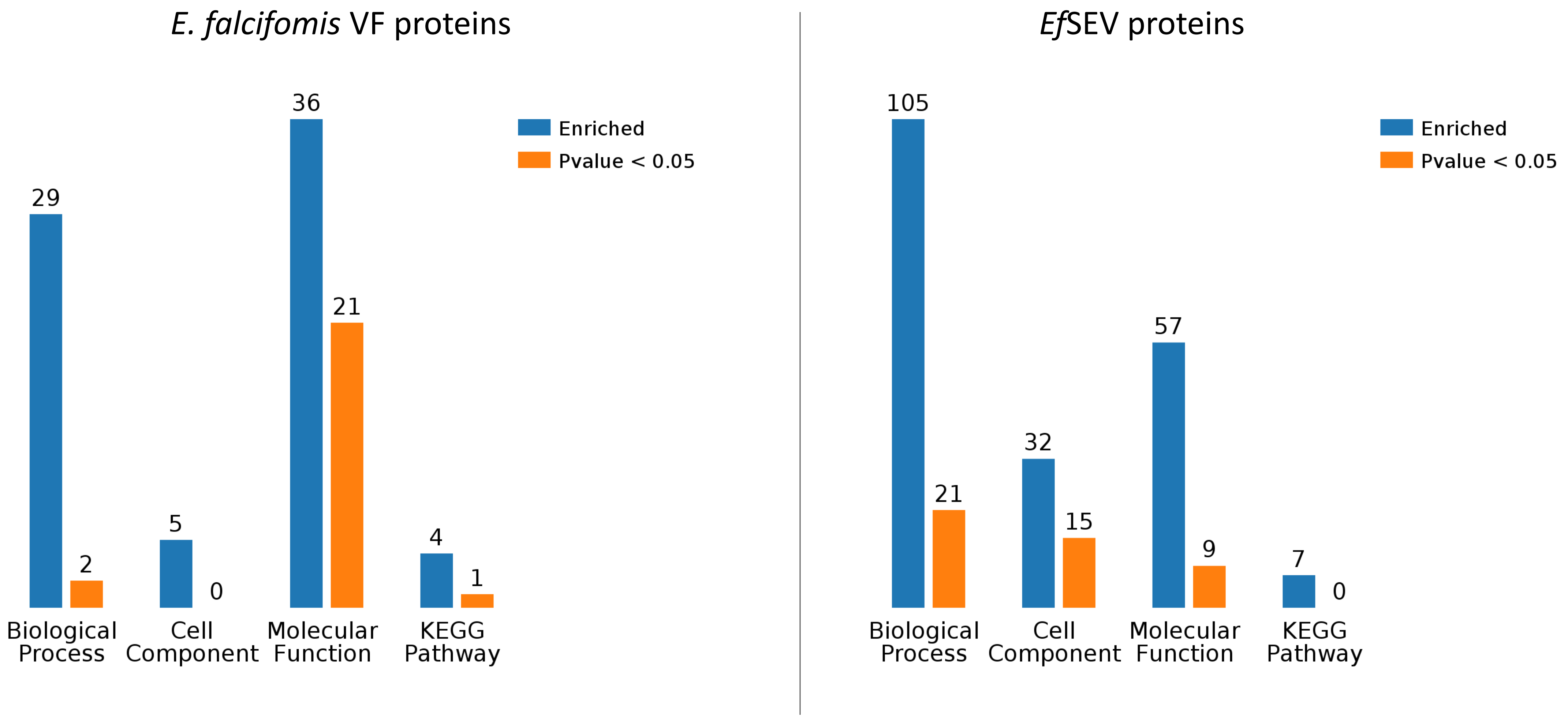

Figure 2-5: statistical summary and KEEG analysis for protein Identified from *E. falciformis* EVs and VF fraction

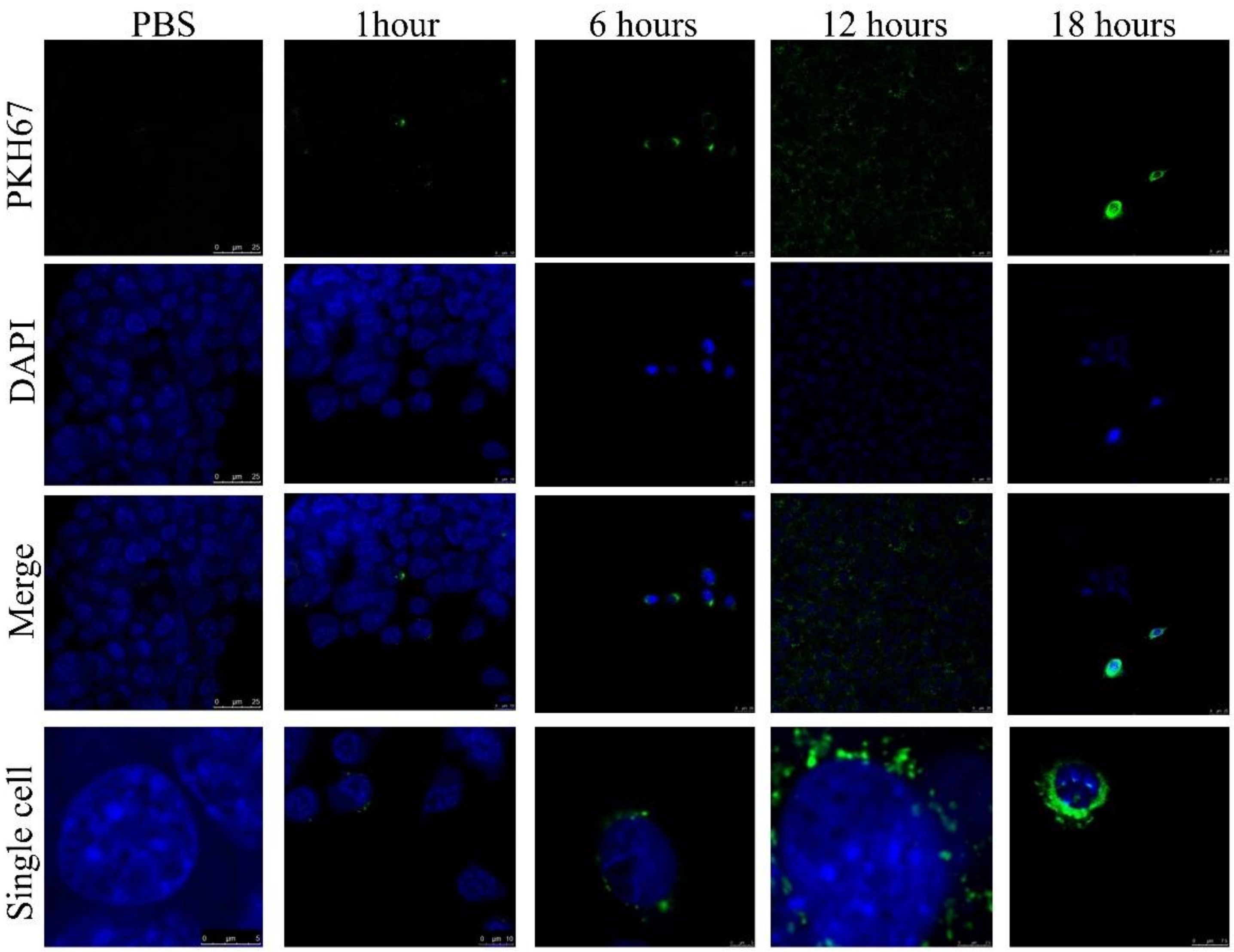

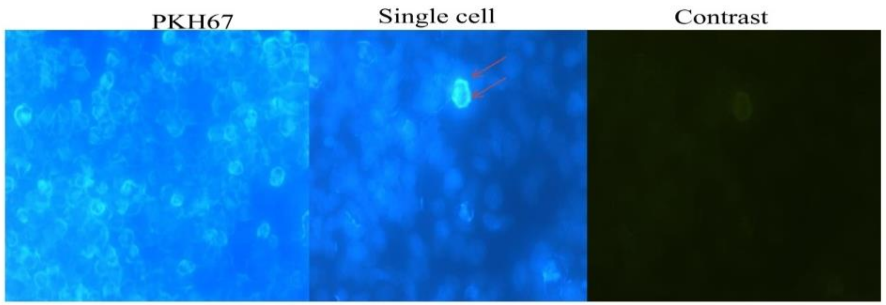

Figure 2-6: Internalization of *Ef*SEVs by murine intestinal epithelial cells. Murine IECs were incubated with green fluorescent PKH67-labellded *Ef*SEVs and processed after 0, 1, 6 and 12 hours for imaging by scanning confocal microscopy; green, labelled EVs; blue, cell nuclei. The negative control was MIECs treated with PBS-PKH67

Table 2-1: Primer sequence for cytokine, chemokine and inflammasome genes

Mouse Gene	Genebank ID	Direction	Sequence (5' to 3')	Expected size	Temp
IL-6	M24221.1	F	GCTTAATTACACATGTTCTCTGGGAAA	93	60
		R	CAAGTGCATCATCGTTGTTCATAC		
mcp1	NM_011333.3	F	AGCCAACTCTCACTGAAG	190	60
		R	TCTCCAGCCTACTCATTG		
IL-1β	NM_008361	F	CCAAAGAAGAAGATGGAAAAACG	113	60
		R	TCTGCTTGTGAGGTGCTGATGTA		
β-actin	NM_007393.5	F	TGGGGTGTTGAAGGTCTC	121	58-61
		R	CTACAATGAGCTGCGTGTG		
IL-18	NM_008360	F	AGGACACTTTCTTGCTTGCCA	183	60
		R	ACAGGCGAGGTCATCACAAG		
casp 11	NM_175362	F	TTTCAAGTCAAGGGGCACGA	233	60
		R	AAGGCATCAAACCCACAGCT		
IL-17	U43088.1	F	CCCTCAGACTACCTCAACCGTTC	192	61
		R	AGGCTCCCTCTTCAGGACCAG		
NLRP6	NM_133946	F	ATACGCGGCTTCTCAGACAAA	237	60
		R	GGTGATGAAGAGCAGGTACACA		
